# Trends and Disparities in Cardiovascular Mortality among HIV-Positive Adults in the United States (2004-2020): A CDC WONDER Database Analysis

**DOI:** 10.2174/011570162X379326251026150021

**Published:** 2026-01-09

**Authors:** Faizan Ahmed, Tehmasp Rehman Mirza, Sherif Eltawansy, Kainat Aman, Sonia Hurjkaliani, Adiesh Sood, Amnah Siddiqui, Yusra Junaid, Momina Siddiqui, Mushood Ahmed, Farman Ali, Aman Ullah, Nisar Asmi

**Affiliations:** 1 Department of Internal Medicine, Duke University Hospital, Durham, NC, USA;; 2 Department of Internal Medicine, Shalamar Medical and Dental College, Lahore, Pakistan;; 3 Department of Internal Medicine, Jersey Shore University Medical Center, Neptune City, NJ, USA;; 4 Department of Internal Medicine, Batterjee Medical College, Jeddah, Saudi Arabia;; 5 Department of Internal Medicine, Dow University of Health Sciences, Karachi, Pakistan;; 6 Department of Internal Medicine, Adesh Institute of Medical Sciences and Research (AIMSR), Bhatinda, India;; 7 Department of Internal Medicine, Karachi Medical and Dental College, Karachi, Pakistan;; 8 Department of Internal Medicine, Rawalpindi Medical University, Karachi, Pakistan;; 9 Department of Internal Medicine, Corewell Health William Beaumont University Hospital, Royal Oak, MI, USA;; 10 Department of Medicine, Mississippi Academy of Sciences, Jackson, United States;; 11 Department of Internal Medicine, University of North Carolina at Charlotte, Charlotte, NC, USA

**Keywords:** HIV, cardiovascular diseases, healthcare disparities, risk factors, epidemiology, mortality

## Abstract

**Introduction:**

A growing link is observed between cardiovascular disease (CVD) and human immunodeficiency virus (HIV), with more results demonstrating a higher CVD incidence among the HIV population. As the life span of HIV patients rises due to the availability of antiviral treatment, more CVDs and their complications keep unfolding.

**Methods:**

This study followed a retrospective cohort study design and implemented the CDC WONDER (Centers for Disease Control and Prevention Wide-ranging Online Data for Epidemiologic Research) platform from 2004 to 2020. It assessed deaths caused by HIV alone and deaths where CVD co-occurred with HIV as per the International Classification of Diseases -10th Revision (ICD-10). The dataset included death certificates from all 50 states and the District of Columbia, involving adults aged 25 years and older. The HIV-related crude and age-adjusted mortality rate (AAMR) per 1,000,000 people was calculated to examine national trends in mortality.

**Results:**

Our study unveiled that CVD and HIV-related deaths reached 50,132 deaths in total, while CVD-related deaths were 24,314,677 in number. The overall age-adjusted mortality rate (AAMR) for CVD and HIV-related deaths among adults decreased from 18.85 (95% CI: 18.23 to 19.47) per 1 million individuals in 2004 to 13.73 (95% CI: 13.27–14.20) per 1 million individuals in 2020, with an average annual percentage change (AAPC) of -2.36 (95% CI: -3.13 to -1.91) (*p* value<0.00001). AAMRs were highest among Black or African Americans, followed by Hispanic or Latinos and Whites, where the AAMR of all the races decreased to variable degrees from 2004 to 2020, with the decrease most pronounced in Black patients.

**Discussion:**

CVD and HIV-related versus CVD-related AAMR varied based on geographical regions, with the highest CVD and HIV mortality observed in the Northeast. Metropolitan areas exhibited higher CVD and HIV-related AAMRs than non-metropolitan areas throughout the study.

**Conclusion:**

Our study highlighted rising mortality rates associated with HIV and CVD-related deaths. These can have multifactorial causes that require prompt investigation. The identification of high-risk communities can provide a general framework for targeted interventions and policies that can mitigate the escalating disease burden and mortality linked with HIV and CVD.

## 1 INTRODUCTION

For the past century, cardiovascular disease (CVD) has been a leading cause of death in the United States [1]. Even though the medical profession has advanced lately, the prognosis of individuals with heart disease is still quite dismal, with high mortality and extensive healthcare expenses. The presence of HIV infection places a person at a higher risk of CVD [2-4]. The recent introduction of antiretroviral therapy has increased survival among people infected with HIV, thereby increasing lifespan and unmasking aetiologies of cardiovascular disease that present early. Baseline studies indicate that the prevalence of heart disease is 1.5-2 times higher among people living with HIV infection at baseline compared to those without the infection [4]. It is predicted that, by 2030, nearly 78% of the patients who test positive for HIV will have heart disease [5, 6]. These significant statistics point towards a greater predicted issue that can be sorted promptly if adequate research paves the path for sufficient interventions. The study of the incidence of HIV and CVD has been greatly aided by earlier research, which frequently concentrated on trends in prevalence, death rates, and demographic differences [7]. To fully comprehend the combined effect of these co-existing conditions, a thorough analysis that compares the mortality trends of CVD alone against those of CVD worsened by HIV infection is needed. The present study aimed to compare mortality trends and disparities of CVD with ramifications in the presence of HIV infection in adults from the United States (US). This analysis has investigated the relationship between HIV and CVD mortality using CDC surveillance data from 2004 to 2020. Our findings can help in better policy-making, informed institutional decisions regarding healthcare, identification of high-risk communities, and targeted interventions that may contribute to healthier national outcomes.

## 2 MATERIALS AND METHODS

### 2.1 Study Design

The data utilized in this study were sourced from death certificates, accessible through the CDC WONDER (Centers for Disease Control and Prevention Wide-ranging Online Data for Epidemiologic Research) platform [8]. The data spanned from 2004 to 2020, focusing on deaths caused by HIV alone and deaths where CVD co-occurred with HIV as per the International Classification of Diseases-10th Revision (ICD-10). Previous studies have steadily demonstrated that these codes have a positive predictive value (PPV) and negative predictive value (NPV) of around 98%, establishing them as a dependable instrument for evaluating this patient group [9]. Similar studies have been conducted, implementing the CDC database to display the correlation between cardiovascular disease and systemic lupus disease [10] or diabetes mellitus [11]. Patients with HIV were identified with *ICD‐10* codes B20-24, and patients with CVD were identified with *ICD‐10* codes I00‐I99 in patients ≥25 years of age. The dataset included death certificates from all 50 states and the District of Columbia; this has also been utilized in previous research to analyze mortality trends across different conditions, with a particular focus on adults aged 25 and older, stratified according to age groups 25-44 years, 45-64 years, and 65-85+ years of age.

The period of 2000-2003 had a visually low number of deaths that felt unnatural to the trend, and a sensitivity analysis was done for the overall trends to understand the impact of including these years on the trend analysis. ICD code stratification revealed that the deaths related to HIV (B20-24) and cardiac arrest (I46) were abnormal in the mentioned years (Fig. **1a**). I46 code was not excluded from the analysis as 45.8% of the deaths that were caused by CVD and HIV were attributed to cardiac arrest (Fig **1b**, **c**).

Institutional review board (IRB) approval was not required because our research study used anonymized publicly accessible government datasets and followed the Strengthening the Reporting of Observational Studies in Epidemiology (STROBE) guidelines for reporting. All data and materials have been made publicly available and can be accessed at https://wonder.c.gov/.

### 2.2 Data Extraction

The study collected data on various mortality-related factors, including population size, year, location of death, demographics, urban-rural classification, regional boundaries, and state-specific categories. Demographic data encompassed age and race/ethnicity. The location of death was categorized into different healthcare settings, such as medical facilities (outpatient, emergency room, inpatient, death on arrival, or unknown status), home, hospice, and nursing homes/long-term care facilities. Race/ethnicity was classified as Hispanic or non-Hispanic White, African American, Asian, or Pacific Islander. These data, derived from death certificates, were previously used in WONDER analysis [12, 13].

The population was categorized using the National Center for Health Statistics Urban-Rural Classification Scheme, which defines urban areas as large central metropolitan, large fringe metropolitan, medium metropolitan, and small metropolitan counties, and non-metropolitan areas as micropolitan and non-core counties, based on the 2013 US Census classification. Additionally, regions were grouped into Northeast, Midwest, South, and West, following the definitions provided by the US Census Bureau. Due to the inherent limitations of the WONDER database, any mortality counts under 10 are withheld to protect the privacy of individuals and are labelled as “suppressed”. In contrast, AAMR of mortality counts under 20 is labelled as “unreliable”.

### 2.3 Statistical Analysis

The HIV-related crude and age-adjusted mortality rate (AAMR) per 1,000,000 people was calculated to examine national trends in mortality. This was accomplished by dividing the total number of deaths in the given year that were related to HIV or CVD and HIV by the population. As previously done, AAMR was calculated by standardizing the HIV-related deaths to the 2000 US population, with 95% confidence intervals (CIs). The Joinpoint Regression Program (Joinpoint V 5.2.0.0, National Cancer Institute, Bethesda, MD, USA) was used to get the annual percent change (APC) with 95% CI in AAMR. AAMRs were used to compare death rates fairly among different populations or historical periods. Mortality patterns were examined using AAMRs. This method identifies significant variations in AAMR over time by applying log-linear regression models where temporal regions occur. The Monte Carlo permutation test calculated APCs with 95% CI for the AAMR at the identified line segments linking the Joinpoints. The weighted average of the APCs was calculated and reported as average APCs (AAPCs) and corresponding 95% CIs to summarize the mortality trend for the entire study period. Statistical significance was inferred based on non-overlapping CIs.

## 3 RESULTS

### 3.1 Annual Trends for CVD and HIV-related Versus CVD-related Age-Adjusted Mortality Rate (AAMR)

The overall age-adjusted mortality rate (AAMR) for CVD and HIV-related deaths decreased from 18.85 in 2004 to 13.73 per 1 million individuals in 2020, with an average annual percentage change (AAPC) of -2.36 (95% CI: -3.13 to -1.91) (*p* value<0.00001) (Fig. **2a**). Notably, the AAMR experienced a significant decline from 2004 to 2011 (APC: -6.47; 95% CI: -10.20 to -4.97) (*p* value=0.0005), followed by another period of decline from 2011 to 2018 (APC: -1.34; 95% CI: -5.00 to 1.09) (*p* value=0.179), and finished off with a striking incline from 2018 to 2020 (APC: 9.43; 95% CI: 1.13-14.37) (*p* value=0.012) (Supplementary Tables **1**, **2**, **3**, **4**).

On the other hand, the overall AAMR for CVD-related deaths among adults decreased from 7092 in 2004 to 6910 per 1 million individuals in 2020, with an AAPC of -0.30 (95% CI: -0.74 to 0) (*p* value=0.053) (Fig. **2b**). Notably, the AAMR experienced a moderate decline from 2004 to 2011 (APC: -2.24; 95% CI: -5.17 to -1.31) (*p* value=0.033), followed by another period of decline from 2011 to 2018 (APC: -0.35; 95% CI: -1.65 to 1.36) (*p* value=0.512), and finished off with a significant incline from 2018 to 2020 (APC: 6.95; 95% CI: 2.57-9.45) (*p*-value < 0.000001) (Supplementary Tables **2**, **3**, **4**, **5**).

Sensitivity analysis was conducted using the Joinpoint regression analysis to assess the validity of using the 2004-2020 study period instead of 1999-2020. For 1999-2020, three distinct trend segments were identified with extraordinarily steep trends; 1999-2021 had an APC of -47.20 (95% CI: –57.82 to –33.47, *p* < 0.0001) and 2001-2004 had an APC 39.44 (95% CI: 19.11 to 60.14, *p* < 0.0001), showing unnatural fluctuations in the dataset (Supplementary Fig. **1a**). Such a rapid fall-and-rise pattern is highly atypical in the context of population-level mortality trends. Consequently, the 2004-2020 study period was used in further stratification analyses, which showed a more natural trend over time (Supplementary Fig. **1b**).

### 3.2 CVD and HIV-related Versus CVD-related AAMR Stratified by Sex

For CVD and HIV-related deaths, adult men (20.43) exhibited higher AAMRs compared to adult women (7.25). Both men and women rates decreased from 2004 to 2020, with the decrease more prominent in women [women: AAPC: -3.64, (CI: -4.52 to -2.87) (*p* value<0.00001); men: AAPC: -1.89, (CI: -2.70 to -1.28) (*p* value<0.00001)] (Fig. **3a**). For adult men, it decreased reasonably from 27.34 in 2004 to 17.48 in 2011 (APC: -6.08; 95% CI: -10.46 to -4.41) (*p*-value < 0.01), and another decline proceeded to 17.42 in 2018 (APC: -0.92; 95% CI: -4.80 to -1.67) (*p* value=0.32), followed by a substantial increase to 21.61 by 2020 (APC: 10.45; 95% CI: 1.65-15.69) (*p*-value < 0.005). Similarly, for adult women, it diminished from 10.78 in 2004 to 6.05 in 2013 (APC: -6.77; 95% CI: -10.05 to -5.72) (*p*-value < 0.0001), followed by an increase to 6.53 by 2020 (APC: 0.54; 95% CI: -2.08-7.60) (*p*-value = 0.579) (Tables **S2**, **S3**, **S4**, **S5**).

For CVD-related deaths, adult men (7514.6) had higher AAMRs compared to adult women (5316.0). Both men and women rates dropped from 2004 to 2020, with the decrease more prominent in women (women: AAPC: -0.54, (CI: -0.96 to -0.26) (*p*-value < 0.002); men: AAPC: -0.23, (CI: -0.72 to 0.14) (*p*-value = 0.138)) (Fig. **3b**). For both cohorts, the decline was observed till 2018, followed by a significant rise till 2020 (APC (2018-2020): men: 7.19, CI: 2.29-9.85, *p* < 0.0001; women: 6.44, CI: 2.26-8.64, *p* < 0.0001) (Tables **S1**, **S2**, **S3**, **S4**).

### 3.3 CVD and HIV-related vs. CVD-related AAMR Stratified by Race/Ethnicity

Due to suppression constraints, racial stratification was based on Hispanics, non-Hispanic Whites, and non-Hispanic Blacks. AAMRs were highest among Black or African Americans (63.2), followed by Hispanic or Latinos (15.2) and Whites (5.8). All the races decreased to variable degrees from 2004 to 2020, with the decrease most pronounced in Hispanics (AAPC: Hispanic: -4.07, CI: -5.35 to -3.23, *p*-value < 0.000001; Black: -3.17, CI: -4.17 to -2.54, *p*-value < 0.000001; White: -0.90, CI: -1.57 to -0.19, *p*-value < 0.01) (Fig. **4a**). A significant incline in APC was observed over different periods for NH White and Hispanic cohorts (APC: White (2011-2020): 2.36, CI: 0.92-4.40, *p*=0.0004; Hispanic (2018-2020): 15.87, CI: 1.22-25.81, *p*=0.02; Black (2018-2020): 9.46, CI: -0.95-15.67, *p*=0.08) (Supplementary Tables **2**, **3**, **5**, **6**).

For comparative reasons, racial stratification was based only on Hispanics, non-Hispanic Whites, and non-Hispanic Blacks for CVD-related deaths. They were highest among Black or African Americans (8285.6), followed by Whites (6257.7) and Hispanic or Latinos (4969.3). The AAMR of the White and Black cohorts decreased, while it increased for the Hispanic cohort from 2004 to 2020. The decrease was more pronounced in the Black cohort (Hispanic: AAPC: 0.35 (CI: -0.40 to 0.90) (*p*-value = 0.24); Black: AAPC: -0.36 (CI: -1.03 to 0.18) (*p*-value = 0.12); White: AAPC: -0.29 (CI: -0.65 to -0.05) (*p*-value < 0.02)) (Fig. **4b**). All cohorts expressed a significant increase from the period 2018-2020 (APC (2018-2020): Hispanic: 17.12 (CI: 8.29-21.94) (*p*-value < 0.00001); Black: 11.02 (CI: 4.03-14.89) (*p*-value < 0.00001); White: 5.53 (CI: 1.89-7.46) (*p*-value < 0.000001)) (Tables **S1**, **S2**, **S3**, **S7**).

### 3.4 CVD and HIV-related Versus CVD-related AAMR Stratified by Age

For CVD and HIV-related deaths, the stratification among age groups showed substantial differences in the AAMR values. Middle-aged adults (45-64 years old) had the highest AAMR of 21.88, followed by older adults (65+ years) with an AAMR of 11.13, and the lowest mortality rates were recorded in younger adults (25-44 years old) with an AAMR of 8.48. Older adults showed the steepest increase in AAMR with an AAPC of 6.63 (95% CI: 3.85 to 4.35) (*p*<0.0001), while the other cohorts had a sharp decline in AAMR; younger adults had an AAPC of -7.60 (95% CI -8.61 to -6.99) (*p*<0.0001) and middle-aged adults with an AAPC of -1.93 (95% CI -2.61 to -1.41) (*p*<0.0001) (Fig. **5a**, Supplementary Tables **2**, **3**, **8**).

For CVD-related deaths, the stratification for age displayed contrasting group findings. Older adults (65+ years old) had the highest AAMR of 26193.6, followed by middle-aged adults (45-64 years) with an AAMR of 2812.4, and the lowest mortality rates were recorded in younger adults (25-44 years old) with an AAMR of 444.4 Younger adults showed the steepest increase in AAMR with an AAPC of 1.34 (95% CI: 0.69 to 1.81) (*p*<0.0001), followed by middle-aged adults with an AAPC of 0.91 (95% CI: 0.34 to 1.37) (*p*<0.0001). Contrarily, the older adult cohort had a decline in AAMR with AAPC of -0.62 (95% CI: -1.04 to -0.33) (*p*<0.0001) (Fig. **5b**, Supplementary Tables **2**, **3**, **8**).

### 3.5 CVD and HIV-related Versus CVD-related AAMR Stratified by Census Regions

On average, over the study period, the highest CVD and HIV mortality was observed in the Northeast (20.25), followed by the South (16.58), West (11.44), and Midwest (5.11). The AAMRs of all regions except the Midwest decreased throughout the study with a most significant decrease in the Northeast (Northeast: AAPC: -5.78 (95% CI: -6.77 to -5.26) (*p*-value < 0.000001); West: AAPC: -1.76 (95% CI: -2.37 to -1.10) (*p*-value < 0.00001); South: AAPC: -1.42 (95% CI: -2.08 to -0.84) (*p*-value < 0.00001); Midwest: AAPC: 1.66 (95% CI: -0.02 to 2.64) (*p*-value = 0.054)) (Fig. **6a**). Midwest showed the most significant increase in APC from 2018-2020, followed by Northeast (APC (2018-2020): Midwest: 17.48, CI: 1.73-27.01, *p*=0.008; Northeast: 12.45, CI: 0.42-19.79, *p*=0.04) (Tables **S2**, **S3**, **S9**).

Furthermore, CVD-related mortality was highest in the South (6524.6), followed by the Midwest (6282.9), Northeast (6204.7), and West (5947.7). The AAMRs of all regions decreased for the study, with the most significant decrease observed in the Northeast (Northeast: AAPC: -0.50 (95% CI: -1.20 to -0.13) (*p*-value = 0.009); West: AAPC: -0.40 (95% CI: -0.78 to -0.09) (*p*-value < 0.01); South: AAPC: -0.32 (95% CI: -0.78 to 0.01) (*p*-value = 0.054); Midwest: AAPC: -0.01 (95% CI: -0.43 to 0.28) (*p*-value = 0.816)) (Fig. **6b**). All cohorts displayed a rise in AAMR from 2018 to 2020, with the highest APC observed in the Northeast, followed by the Midwest, South, and West (Supplementary Tables **2**, **3**, **9**).

### 3.6 CVD and HIV-related versus CVD-related AAMR Stratified by Urbanization

With regards to urbanization, metropolitan areas (14.89) exhibited higher CVD and HIV-related AAMRs than non-metropolitan areas (6.48) throughout the study period. The AAMR of both metropolitan and non-metropolitan areas decreased from 2004 to 2020, with a more significant decrease in metropolitan areas (metropolitan: AAPC: -2.57 (CI: -3.37 to -2.09) (*p*-value < 0.00001); non-metropolitan: AAPC: -0.21 (CI: -1.13 to 0.73) (*p*-value = 0.67)) (Fig. **7a**, Supplementary Tables **2**, **3**, **10**).

On the contrary, CVD-related AAMRs were lower in metropolitan areas (6129.1) than in non-metropolitan areas (7088.6). The AAMR of metropolitan areas decreased from 2004 to 2020, while non-metropolitan areas experienced an increase in the same period (metropolitan: AAPC: -0.43 (CI: -0.93 to -0.08) (*p*-value = 0.026); non-metropolitan: AAPC: 0.15 (CI: -0.25 to 0.42) (*p*-value = 0.356)) (Fig. **7b**). Furthermore, parallel to the majority of the cohorts, metropolitan and non-metropolitan areas had a significant increase from 2018-2020 (metropolitan: APC: 6.58 (CI: 1.56-9.20) (*p*-value < 0.00001); non-metropolitan: APC: 6.49 (CI: 2.48-8.70) (*p*-value < 0.00001)) (Tables **S2**, **S3**, **S10**).

Between 2004 and 2020, CVD and HIV-related deaths reached 50,132 deaths in total among adults aged 25+ years in the United States. These fatalities were distributed across various settings, with most occurring in medical facilities (62.5%), 18.9% at the decedents’ homes, 10.4% in nursing homes/long-term care facilities, 4.4% in hospice facilities, and 3.8% at other locations. Over the same period and population, CVDs accounted for 24,314,677 deaths. In accordance, most deaths occurred in medical facilities (42.7%), 26.6% at the decedents’ homes, 21.9% in nursing homes/long-term care facilities, 4.1% in hospice facilities, and 4.7% at other locations (Supplementary Table **11**).

### 3.7 CVD and HIV-related Versus CVD-related AAMR Stratified by State

For CVD and HIV-related deaths, states falling within the top 90th percentile included the District of Columbia (97.81), New York (38.49), Georgia (27.00), Florida (25.80), and Mississippi (24.77). In contrast, states in the lower 10th percentile included Iowa (2.57), Montana (2.57), Wisconsin (2.33), New Hampshire (2.18), and Idaho (1.94). North Dakota and Wyoming had unreliable data (Fig. **8a**, Supplementary Tables **12**, **13**). On the other hand, with respect to CVD-related deaths, states falling within the top 90th percentile included Mississippi (8898.8), Oklahoma (8228.0), West Virginia (7840.7), Arkansas (7580.8), and Alabama (7552.4). In contrast, states in the lower 10th percentile included Colorado (5332.9), Minnesota (5303.3), Utah (5245.0), Arizona (5177.1), and Hawaii (5097.3) (Fig. **8b**, Supplementary Tables **12**, **13**, **14**) (Supplementary Figs. **2**, **3**, **4**).

### 3.8 CVD and HIV-related Versus CVD-related AAMR with Respect to the Impact of Coronavirus Disease-2019 (COVID-19)

**Fig. (9):**
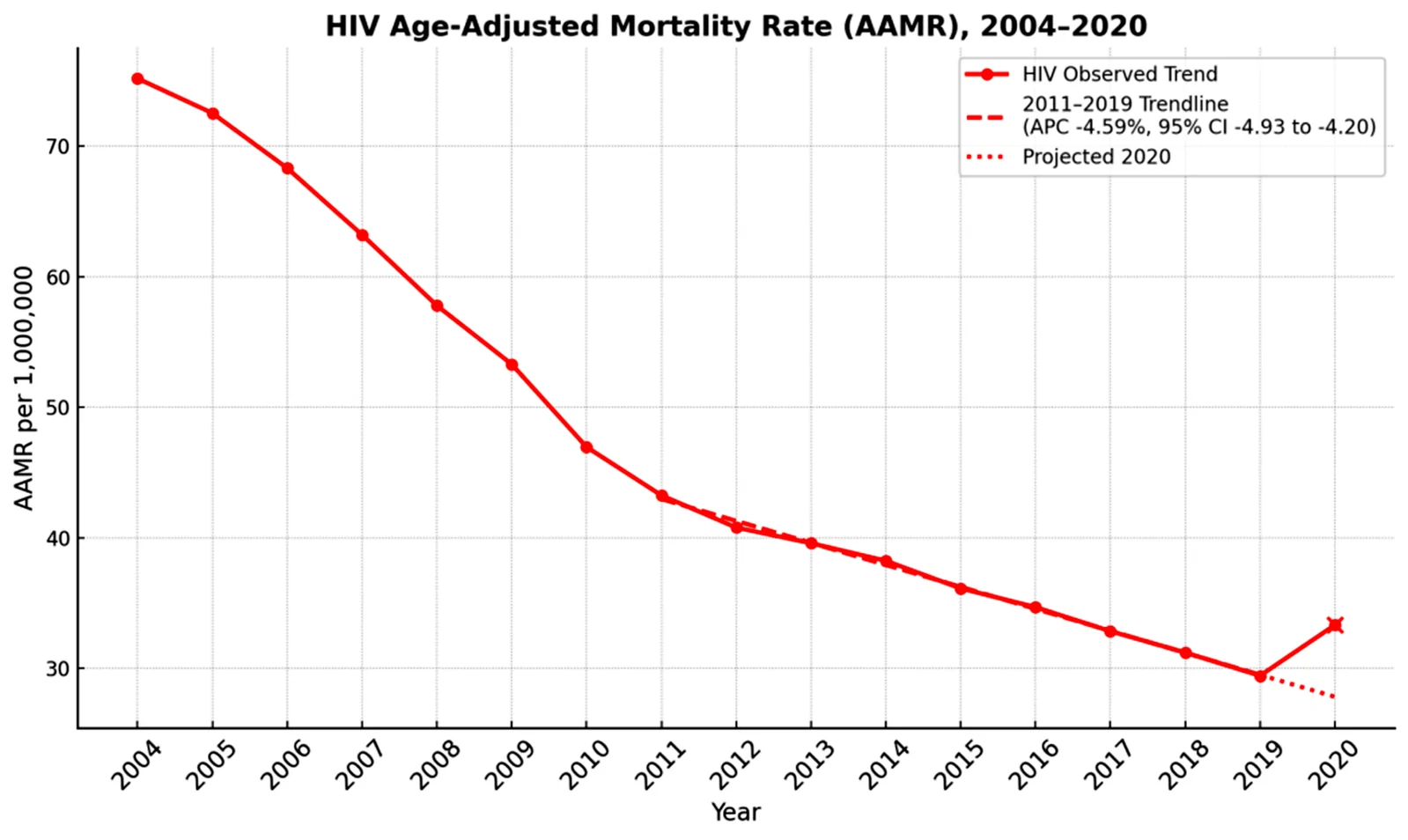
HIV-related AAMRs in the US (2004-2020), highlighting the incline in 2020, related to COVID-19.

**Fig. (10):**
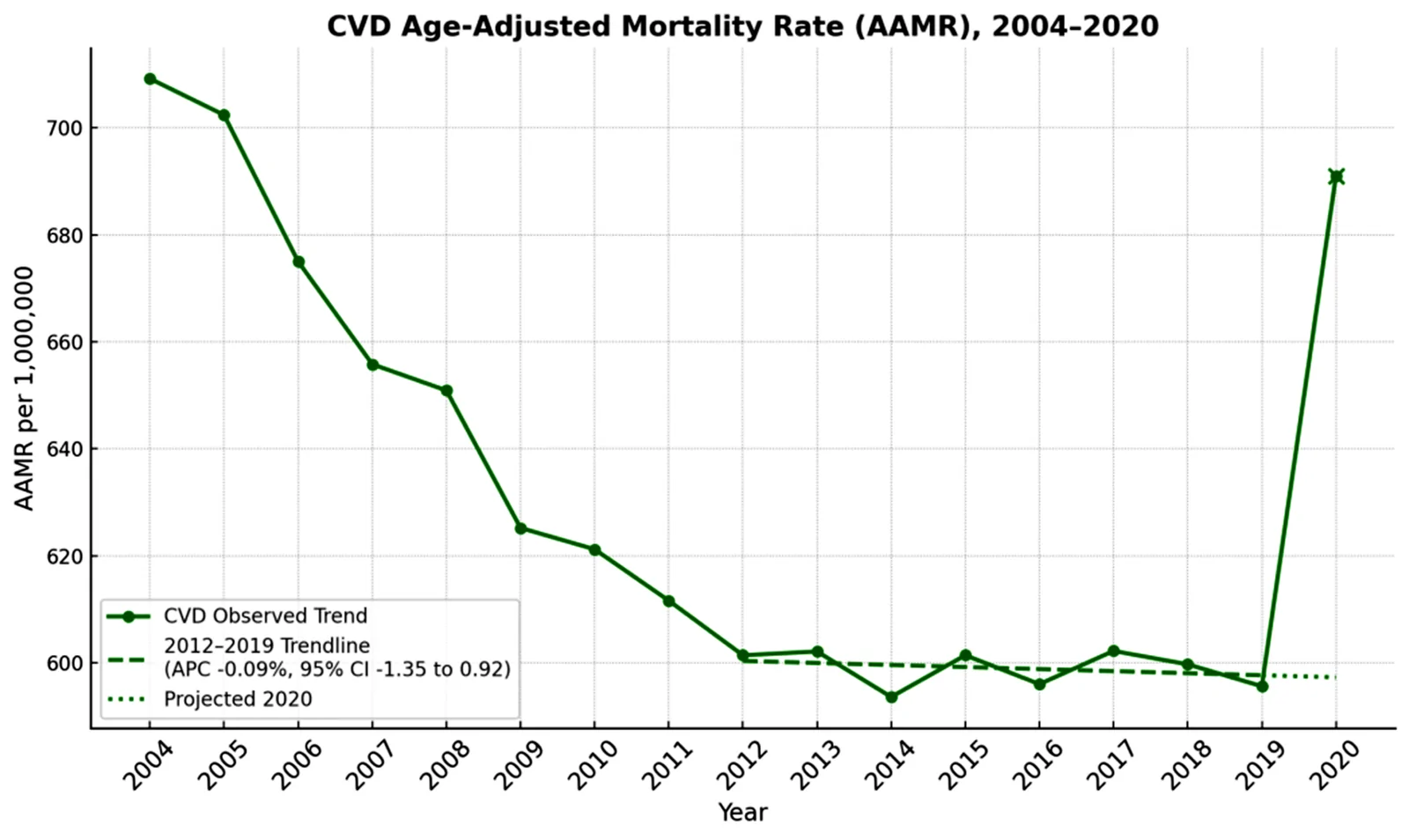
Cardiovascular disease-related AAMRs in the US (2004-2020), highlighting the incline in 2020, related to COVID-19.

**Fig. (11):**
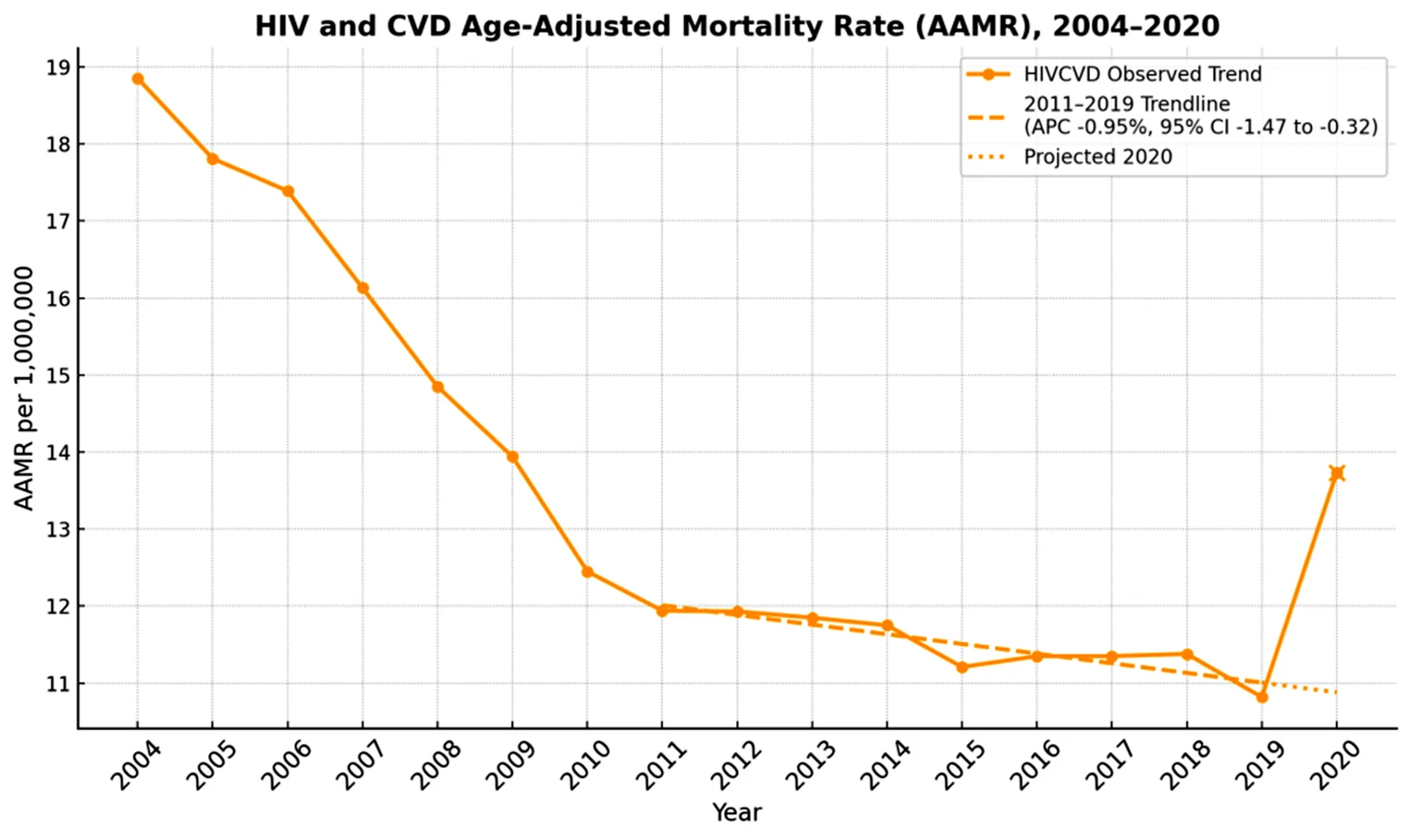
HIV and cardiovascular disease-related AAMRs in the US (2004-2020), highlighting the incline in 2020, related to COVID-19.

The overall AAMRs given for CVD and HIV-related deaths, as well as CVD-related deaths, have been explained above. There was a significant incline in the latter years of our study, *i.e*., from 2019-2020. This sharp rise in mortality can be explained by COVID-19 and its impact. The total number of deaths caused by HIV in the year 2019 was 2703, and in 2020, this value increased to 3511. The trend would have largely remained similar to the years before if not for the pandemic. This has been illustrated in Figs. (**9**, **10**, and **11**). The total number of deaths in the year 2020 attributed to COVID-19 and HIV was 869, with an AAMR value of 3.30 (95% CI: 3.08-3.53) (Figs. **9**, **10**, and **11**).

## 4 DISCUSSION

Over 1.2 million people in the United States are living with HIV [14], signifying a worrying trend. Studies reveal that people with HIV are much more likely to get heart disease than the rest of the population [15, 16]. The study also demonstrates a 1.5 to 2 times higher risk of diseases, like heart failure and heart attacks [17, 18]. This is especially concerning because healthcare providers could notice progress in treating HIV. Even though the HIV prognosis is achieving a promising change, heart disease is still a significant threat to people living with the virus [19].

Research indicates that HIV patients often have ongoing cardiac complications impacting the left ventricle, varying from mild left ventricular systolic dysfunction to severe diastolic dysfunction [20]. Other cardiomyopathies, including but not limited to myocardial infarction and heart failure, are also linked with HIV [21, 22]. Interestingly, recent data indicate that HIV infection nearly doubles the risk of acute heart failure, even in people without a history of coronary heart disease [23]. There is also a connection between a low CD4 count and a higher risk of heart attacks. A lower CD4 to CD8 ratio is also linked to an increased risk of coronary atherosclerosis [24, 25]. This increased risk can also be attributed to the adverse effects of HIV therapy [26]. The biochemical manifestations of HIV can also be a significant cause, as it leads to vascular dysfunction due to cytokine and endothelial activation; the underlying lifestyle factors can also be a pivotal cause of increased CVDs associated with HIV [27-29].

Women and men with HIV seem to be affected differently [30]. Gender discrepancies exist, with men regularly having higher age-adjusted death rates than women. However, the decline in female death rates is a positive trend. Female death rates dropped from 10.78 per 1,000,000 persons in 1999 to 6.53 by 2020. Male death rates fell initially from 27.34 to 17.42 per 1,000,000 individuals, but by 2020, they had risen to 21.61 per 1,000,000. Males were more variable and had consistently higher death rates than females, highlighting the continuing gender disparity. In HIV-infected persons, women had a considerably higher relative risk of cardiovascular disease than men (1.89) compared to controls (standardised incidence ratio: 2.91) [31]. Another study revealed that women were less likely than men to get cardiovascular disease interventions, such as invasive procedures, lipid-lowering drugs, and antihypertensive medications [32]. Interestingly, new studies demonstrate that women with HIV appear to be at a considerably higher risk of heart disease, 1.5 to 2 times higher compared to both men with HIV and those without HIV at all [33]. This might be due to several factors, including higher obesity rates and metabolic syndrome in women with HIV, along with potential shortcomings in managing cholesterol and blood pressure [34]. Sex hormones and how fat is distributed might also play a role [35].

HIV is a disease that affects the socially marginalised communities disproportionately, including racial and geographical differences [36]. Race plays a significant factor, with Black Americans having the highest death rates (826.8 per 100,000), followed by Whites (625.8) and Hispanics or Latinos (469.5). Black men and women seem to be most affected by having multiple health conditions at once, including heart disease, obesity, hypertension, diabetes, and kidney disease [37-41]. They have a higher risk of HIV-related CVDs [42]. Several factors have been linked to these findings, including the socioeconomic disparities; access to healthcare and health insurance are some of them [43]. The burden of HIV and CVD on the underrepresented racial and ethnic groups can be managed by an early referral to a cardiologist; this depends on various factors, including access to healthcare facilities [44]. Black adults with HIV have reported increased prevalence of cardiovascular symptoms, including chest palpitations, chest pain, and shortness of breath, as compared to other races [45]. These findings highlight the need for targeted interventions and resources to address these inequalities.

There are geographic disparities in cardiovascular mortality among HIV-positive individuals as well [46, 47]. Deaths from heart disease among HIV patients are on the rise in the South [48]. This is consistent with our research findings, which reported the highest rates in the Northeast, followed by the South. The lack of healthcare facilities in the affected areas adequately explains these differences in the geographical distributions. Suboptimal geographical accessibility to HIV care is an important structural barrier in the US, disproportionately affecting rural communities [49]. HIV-affected populations in underserved communities are more likely to be affected by the negative impacts, including HIV- related stigma, poverty, homelessness, and lack of insurance. These are significant barriers to accessing HIV care and getting timely treatment, hence increasing the disparities [50, 51]. Rural populations are less likely to be tested and treated for HIV due to the distance to the nearest HIV care facility [52]. Delayed HIV care has also been described due to psychological distress, fear, substance use, and lack of information, food, and transport [53]. HIV-related discrimination, depression, and lack of social support are contributory factors to this increasing disparity [54].

The consistent downward trend in the HIV-related mortality has been reassuring for the masses and policymakers alike. Still, the sudden surge in the incidence of deaths reported in recent years is a cause of concern. The increase in mortality can be attributed to several causes, but the most prevalent and relevant could be the COVID-19 pandemic [55]. Increased deaths were reported across all age groups with significant racial and geographical disparities [56, 57]. The interplay between the two diseases remains complex, as the synergistic effect leads to reported increased mortality. The exacerbation of other chronic conditions due to COVID-19 may also contribute. Another valuable insight could be the summation effect caused by multiple diseases that severely compromise the immunity, *i.e*., HIV and COVID-19. This immunosuppressed state further contributes to the increased mortality levels reported in the latter years of our study.

According to the age group-stratified analysis, the highest mortality was observed between the ages of 45 and 64 years. This adult population is at a greater risk for HIV-associated CVDs and consequential mortality. This could be due to the increased life span in HIV owing to compliance with antiretroviral therapy. The treatment for HIV prolongs the life expectancy and hence increases the incidence and prevalence of chronic diseases, including CVDs. Our findings demonstrated that over half (around 52%) of the deaths occurred in hospitals, which emphasizes the importance of hospitals for end-of-life care. At the same time, many patients expired at home or in long-term care facilities, which could be linked to patient preferences regarding comfort measures.

The escalating mortality associated with HIV and CVD in recent years is a cause of immense concern. Urgent policies are essential to alleviate the impending burden of disease. Some of the steps that can be taken include the following: highlighting the underserved areas and identifying the gaps in access to healthcare; providing education and awareness regarding HIV in hopes to reduce the stigma and increase social support; ease of access to healthcare facilities and HIV care centres primarily in the vulnerable populations; provision of necessities to combat the structural causes of lack of compliance to healthcare, including food and transport; use of mobile healthcare services that can periodically visit the rural areas and monitor disease progression; and making insurance policies easier for low income communities. These measures can ensure proper disease follow-up and treatment, reducing the adverse effects and mortality rates.

Overall, these data indicate that a comprehensive and multi-pronged approach is required to tackle the rising incidence of HIV patients and the associated CVDs. This means creating programs that take into account gender and cultural backgrounds and directing resources toward the most efficient approaches. Our study has identified high-risk populations threatened by increasing mortality rates that could benefit from timely targeted interventions. These communities should be served adequately by providing essential health facilities. Efforts should be made to overcome the social and structural barriers to HIV and associated symptoms, including CVD. This can help improve the overall national health outcomes.

## CONCLUSION

This study has provided insights into the temporal trends and disparities in the death rates from CVDs occurring in HIV in the US. The geographic disparities emphasize the necessity for regional healthcare initiatives. Rural and non-metropolitan areas usually encounter significant healthcare resource constraints and require improved medical infrastructure and specialized healthcare providers. The identification of high-risk communities highlights a substantial trend in disparities that needs to be addressed promptly. Healthcare policies and targeted interventions on time are essential to mitigate the escalating burden of HIV and CVD-related mortality in the US.

## STUDY LIMITATIONS

The study encountered several limitations that warrant consideration. Primarily, it relied on the CDC WONDER database, which uses cause‐specific mortality using ICD codes obtained from information reported in death certificates; therefore, this may be subject to misclassification and coding errors. Potential errors in this dataset might have skewed the findings. Causality cannot be established due to the retrospective cohort; only association can be reported. The interpretation of trends might have been influenced by unreported factors, leading to biases in the outcomes. As the CDC does not provide information for counties with event counts <20, we could not examine data from all county-equivalent regions in the US. Places with <10 deaths in the CDC WONDER system were excluded due to data confidentiality, which may cause missing data, especially in rural areas. Furthermore, the exclusion of medical details, such as particular biological indicators, HIV stage or cluster of differentiation (CD)-4 count, viral load, therapeutic protocols, diagnostic results, and treatment methods, significantly hampered the analysis of the rise in mortality rates. Also, the lack of extensive subgroup evaluations led to a lack of understanding, hindering the discovery of root causes for the escalating death rates. Additionally, the anomalous data for 2000-2003 limited the trend analysis to only 2004-2020. Lastly, influential social and economic determinants of health, including comorbidities, lifestyle factors or risk factors, along with data on medical interventions, access to treatment, and quality of care, were omitted, further limiting the scope of the study. Finally, important risk factors, such as smoking, obesity, diabetes, and hypertension, were not controlled for in the analyses.

## Figures and Tables

**Fig. (1a):**
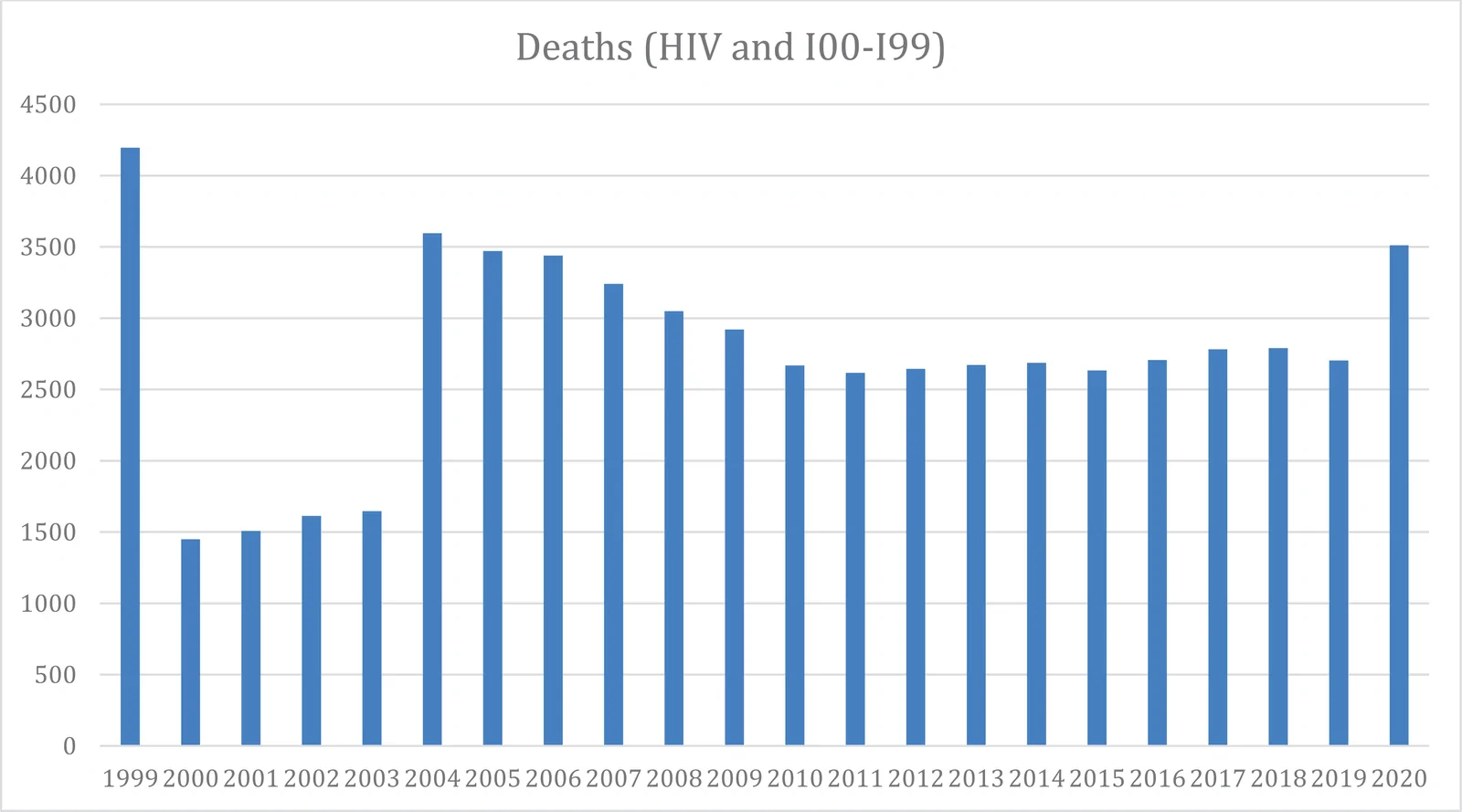
Overall cardiovascular disease and HIV-related deaths in the United States (1999 to 2020).

**Fig. (1b):**
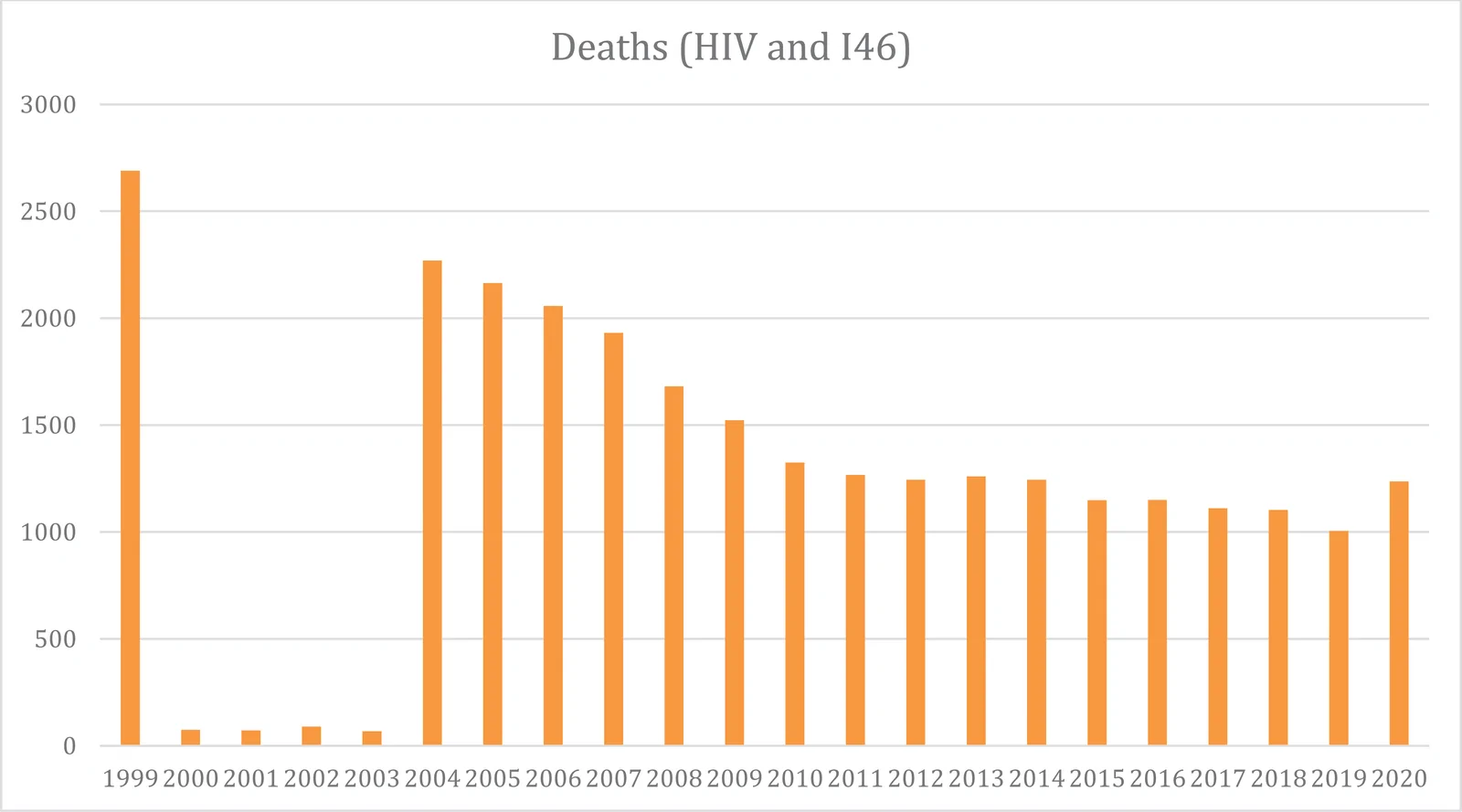
Overall cardiac arrest and HIV-related deaths in the United States (1999 to 2020).

**Fig. (1c):**
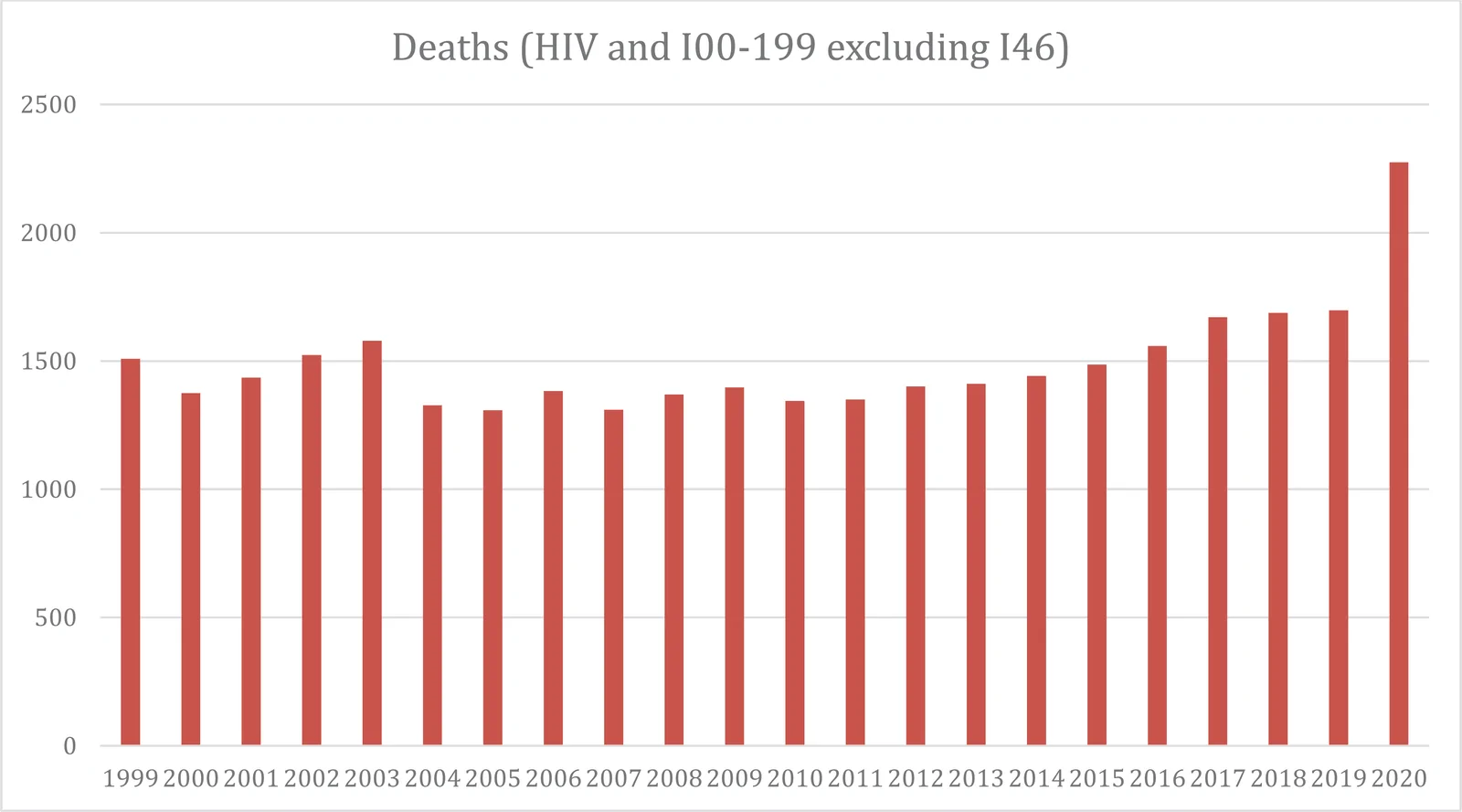
Overall CVDs, excluding cardiac arrest, and HIV-related deaths in the United States (1999 to 2020).

**Fig. (2a):**
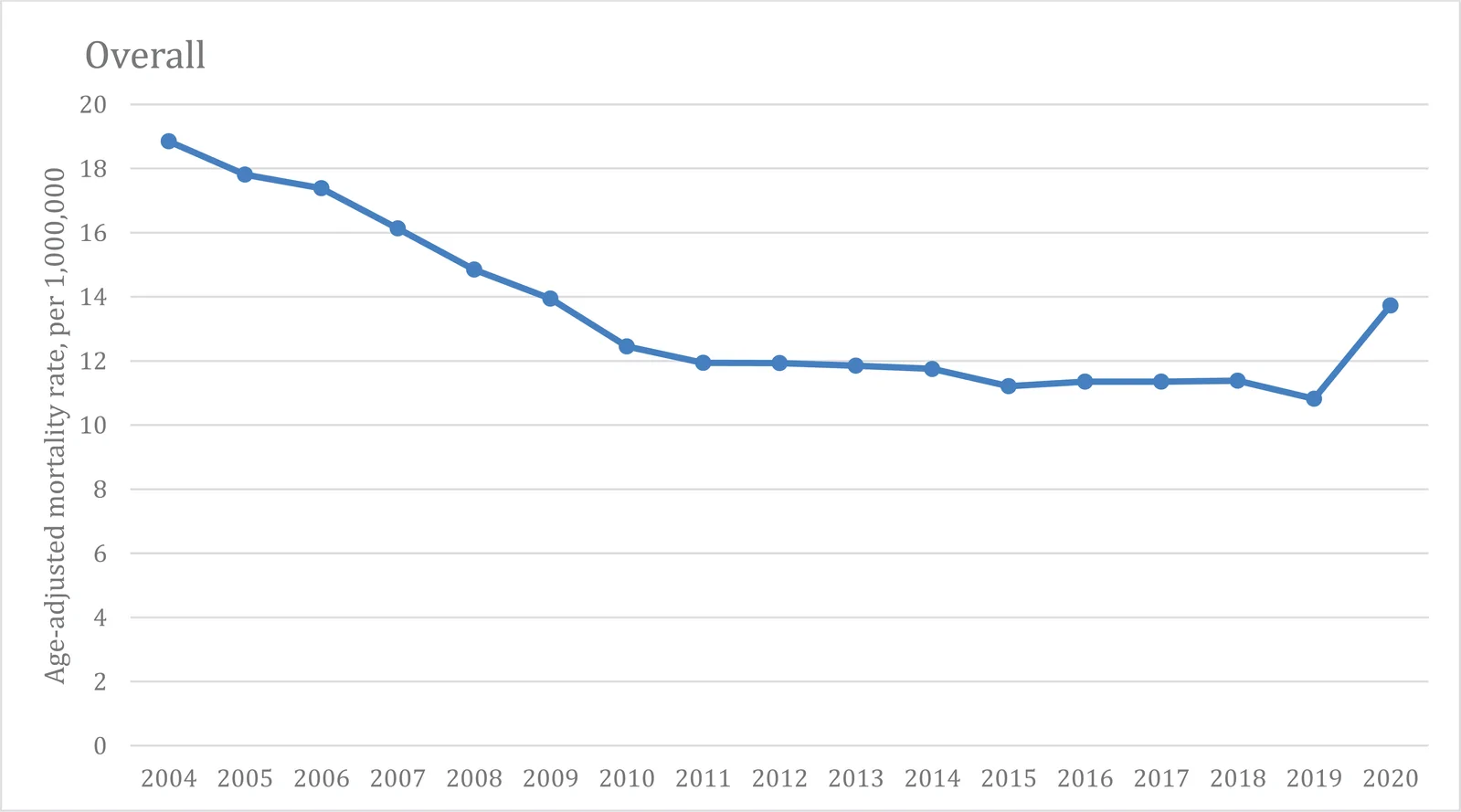
Cardiovascular disease with HIV-related AAMRs per 1,000,000 in the United States (2004 to 2020).

**Fig. (2b):**
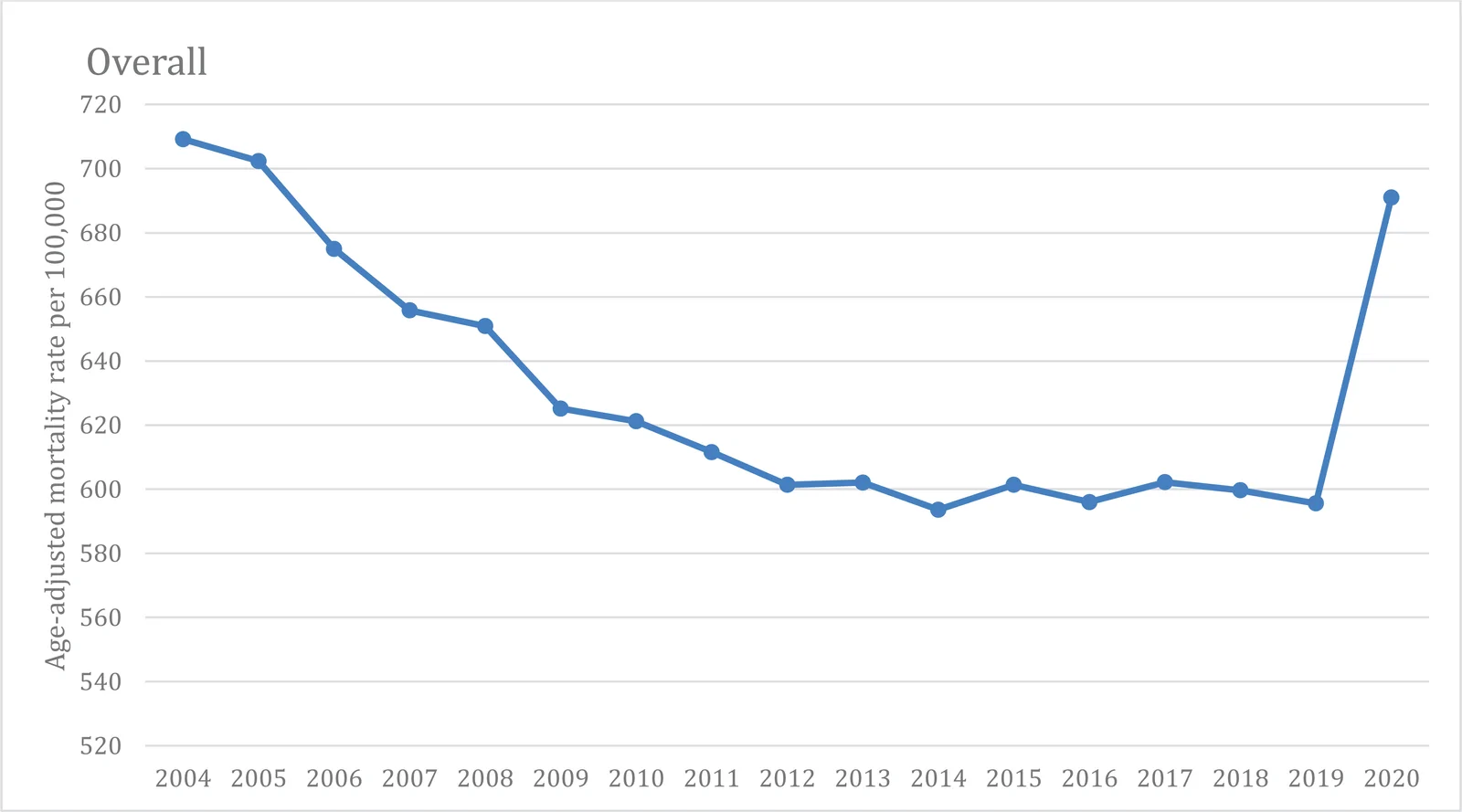
Overall cardiovascular disease-related AAMRs per 1,000,000 in the United States (2004 to 2020).

**Fig. (3a):**
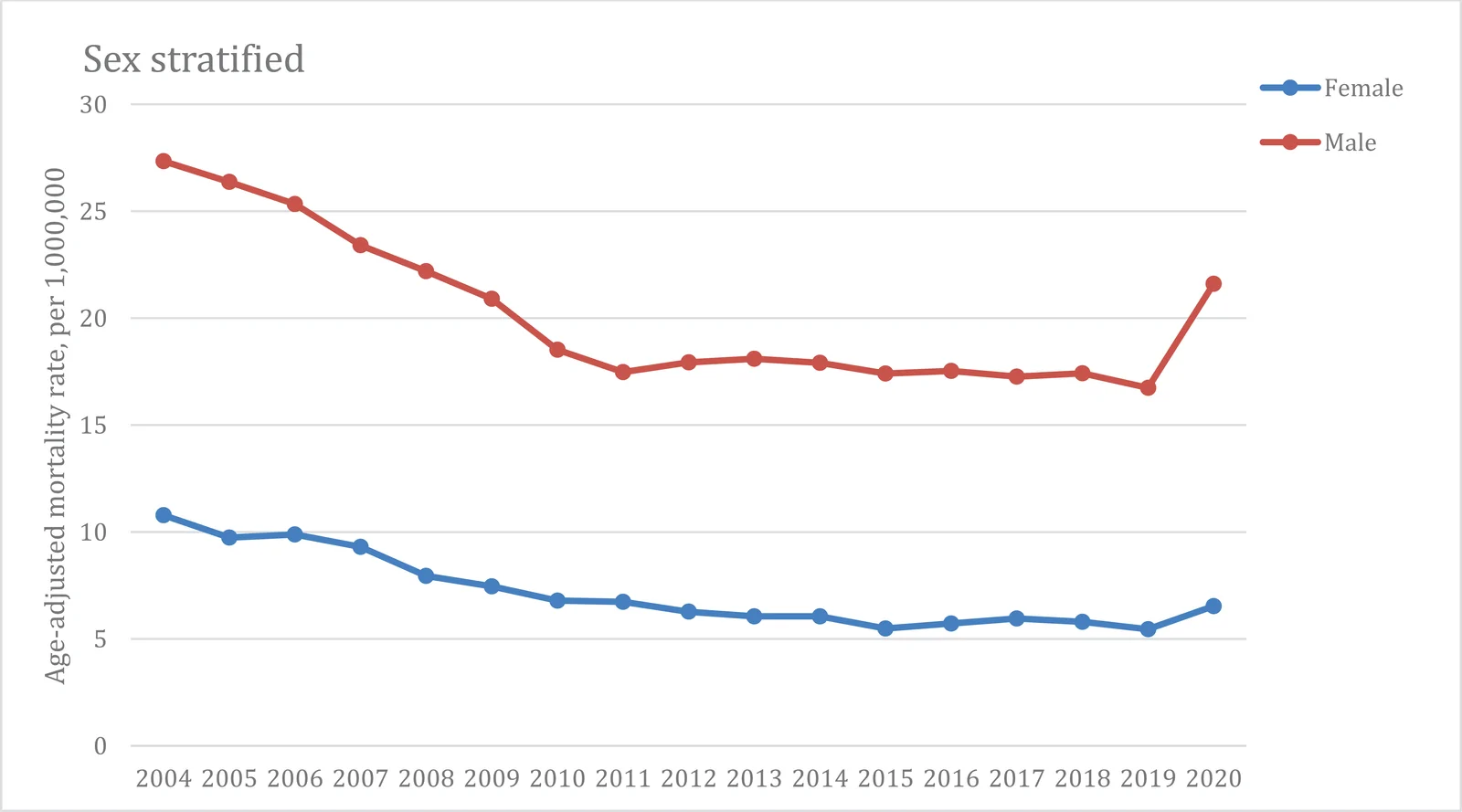
Sex-stratified cardiovascular disease with HIV AAMRs per 1,000,000 in the United States (2004 to 2020).

**Fig. (3b):**
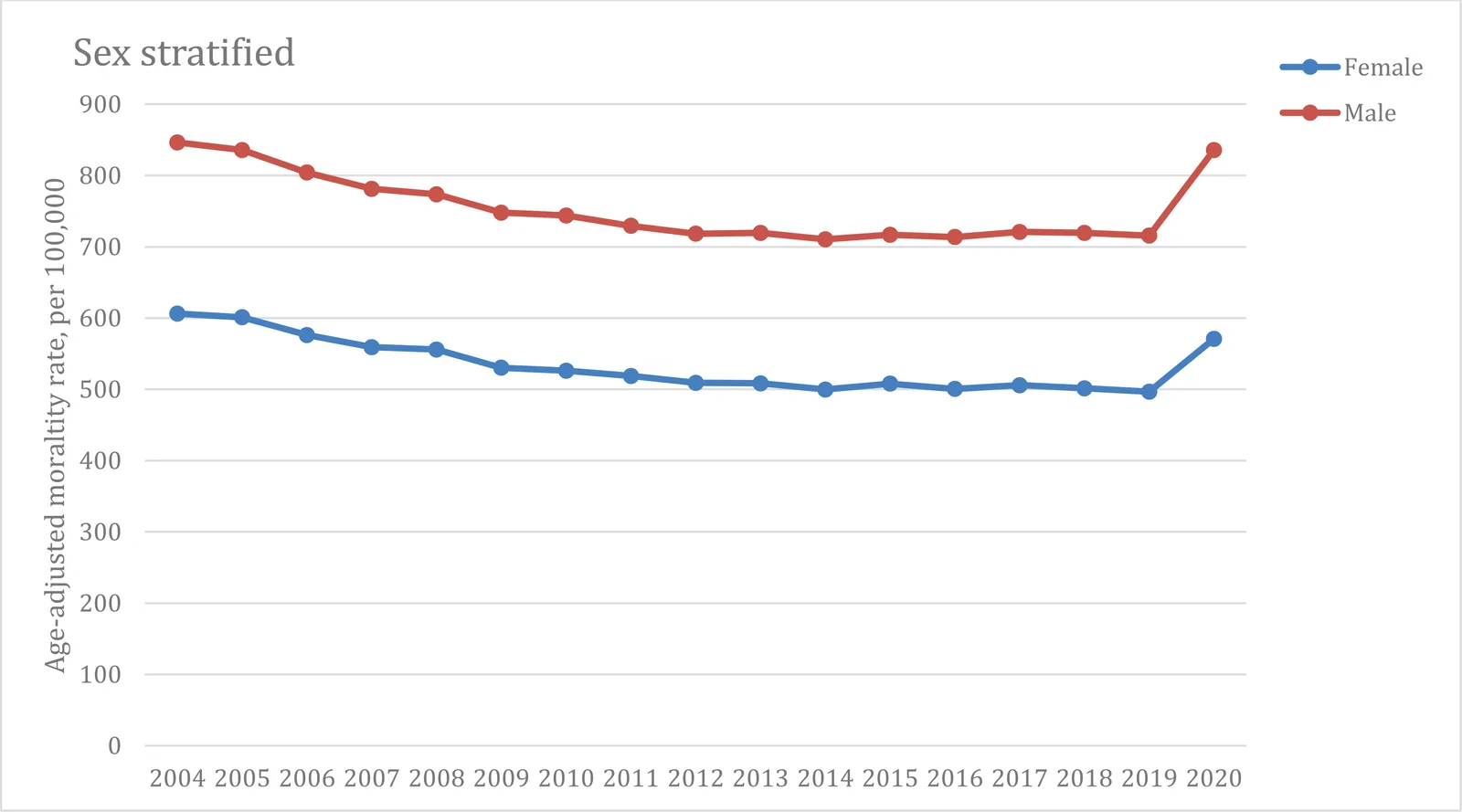
Sex-stratified cardiovascular disease-related AAMRs per 1,000,000 in the United States (2004 to 2020).

**Fig. (4a):**
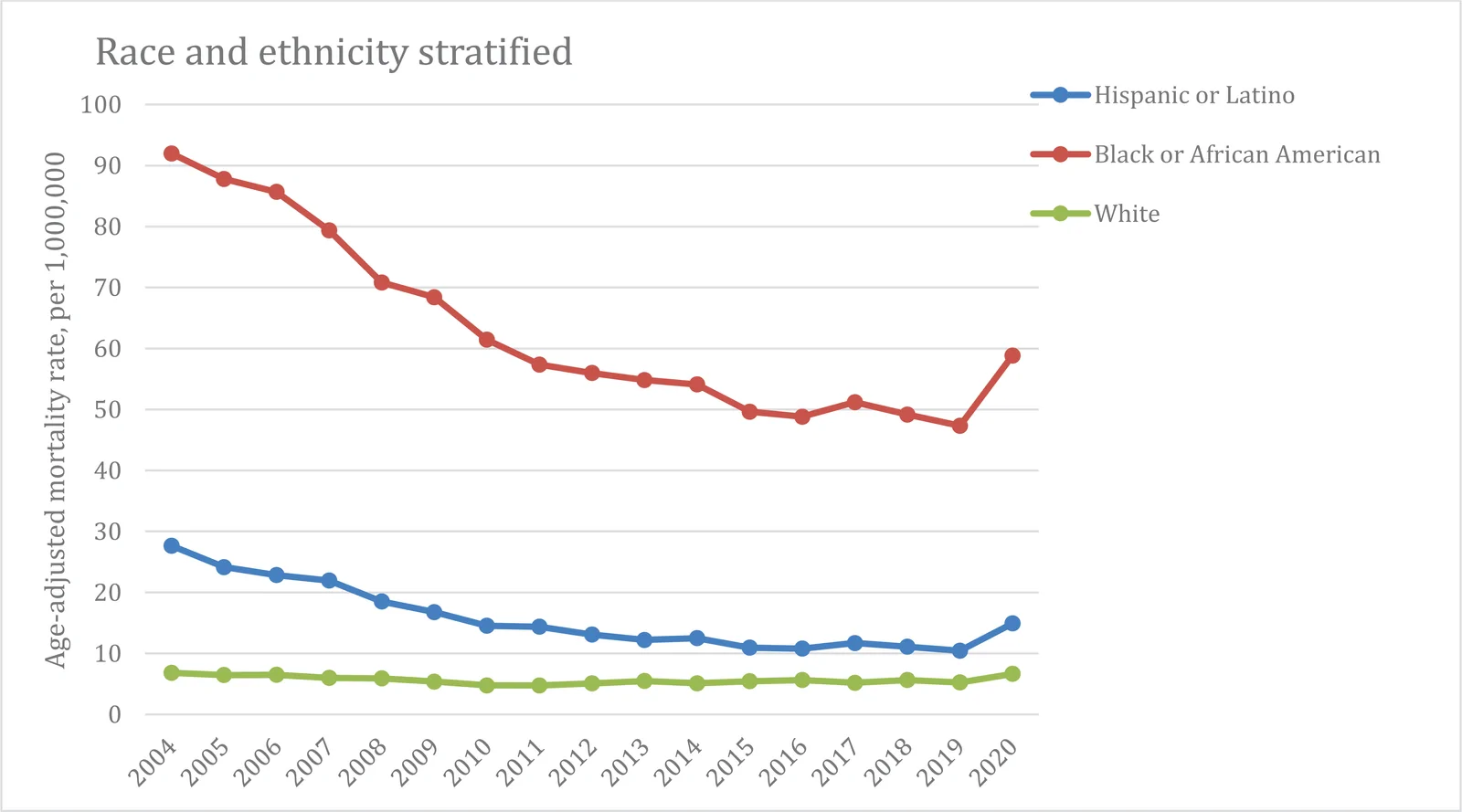
Cardiovascular disease with HIV-related AAMRs per 1,000,000 deaths for each race (Hispanics, Whites, and Blacks) in the United States (2004 to 2020).

**Fig. (4b):**
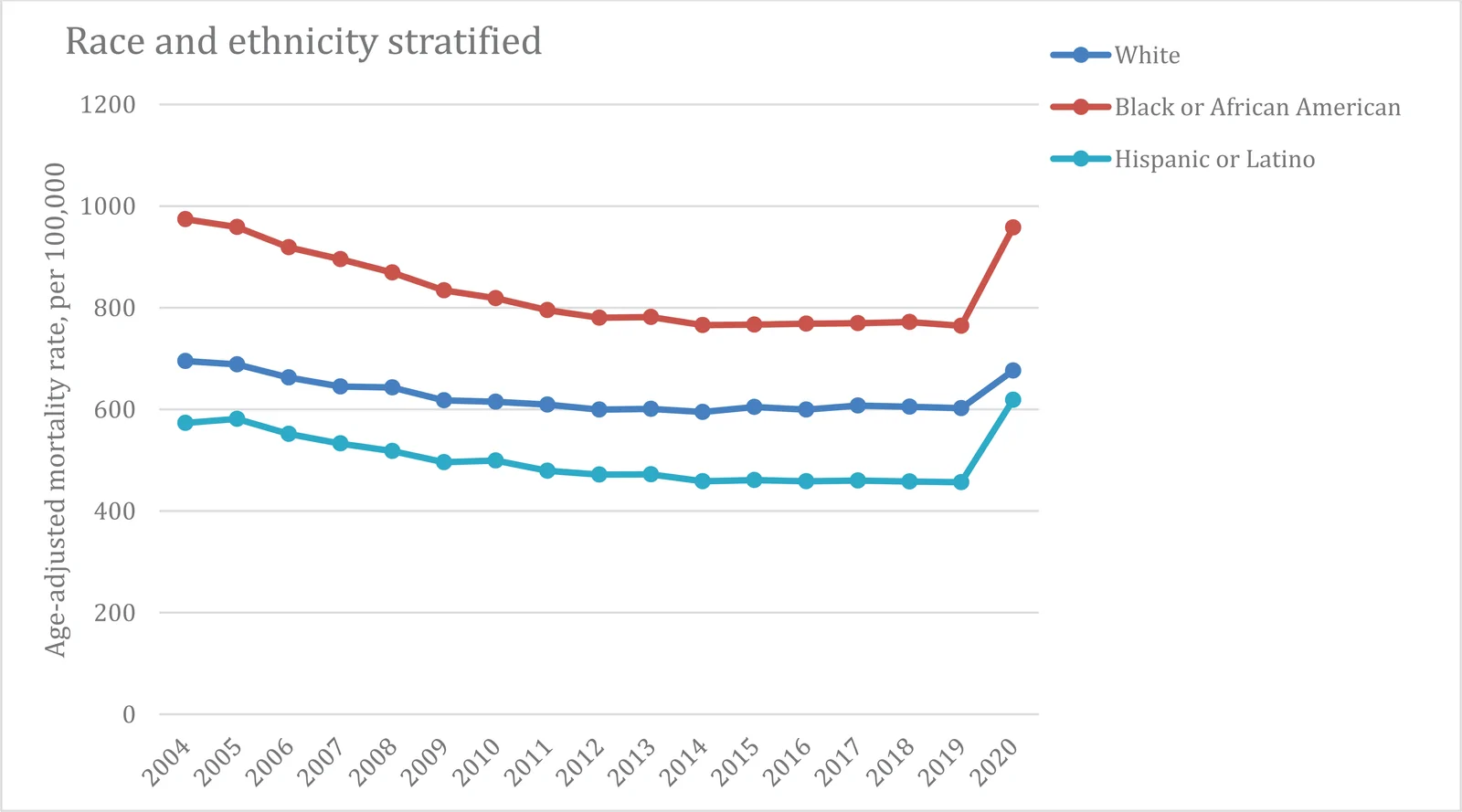
Cardiovascular disease-related AAMRs per 100,000 deaths for each race (Hispanics, Whites, and Blacks) in the United States (2004 to 2020).

**Fig. (5a):**
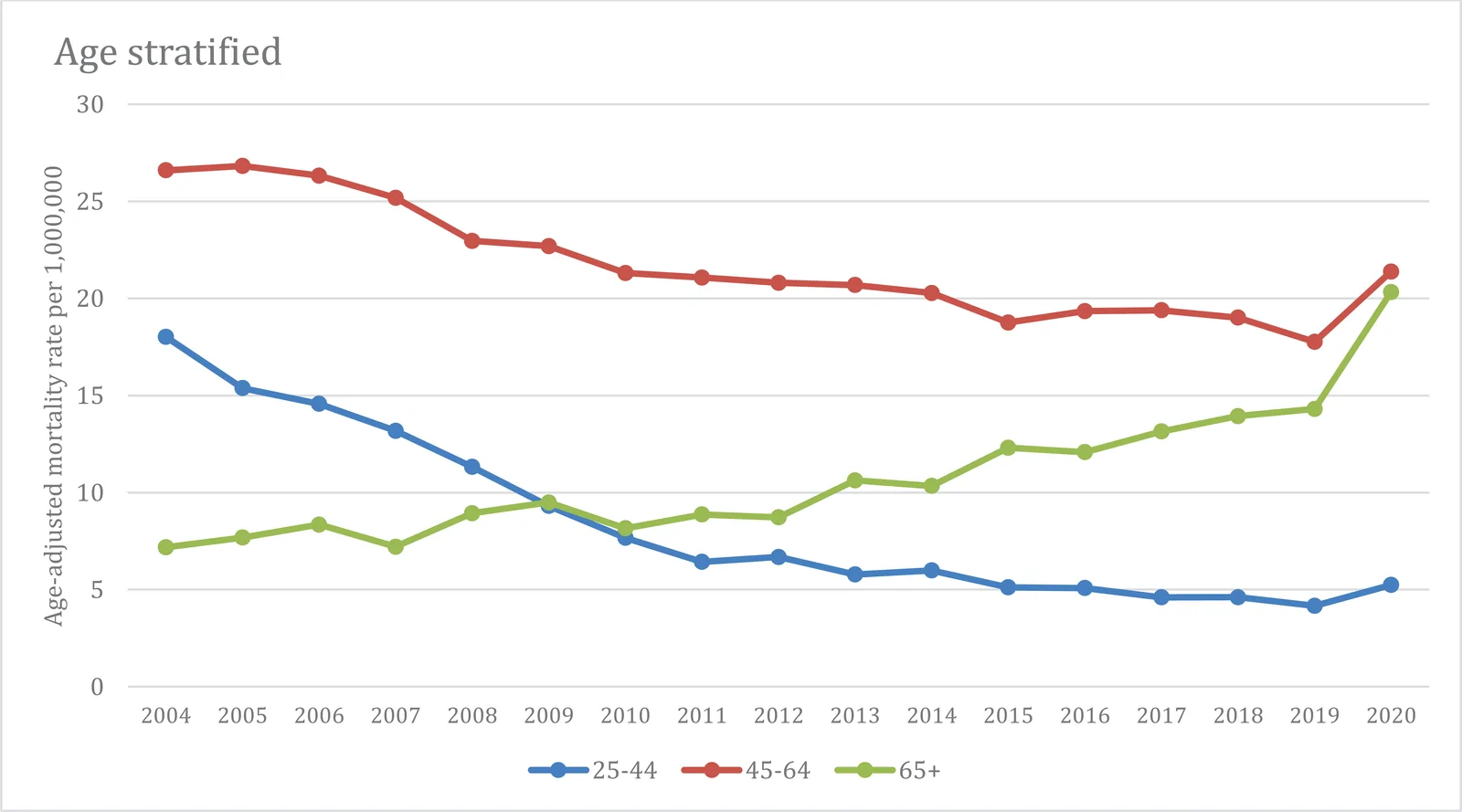
Cardiovascular disease and HIV-related AAMRs per 1,000,000 deaths stratified by age in the United States (2004 to 2020).

**Fig. (5b):**
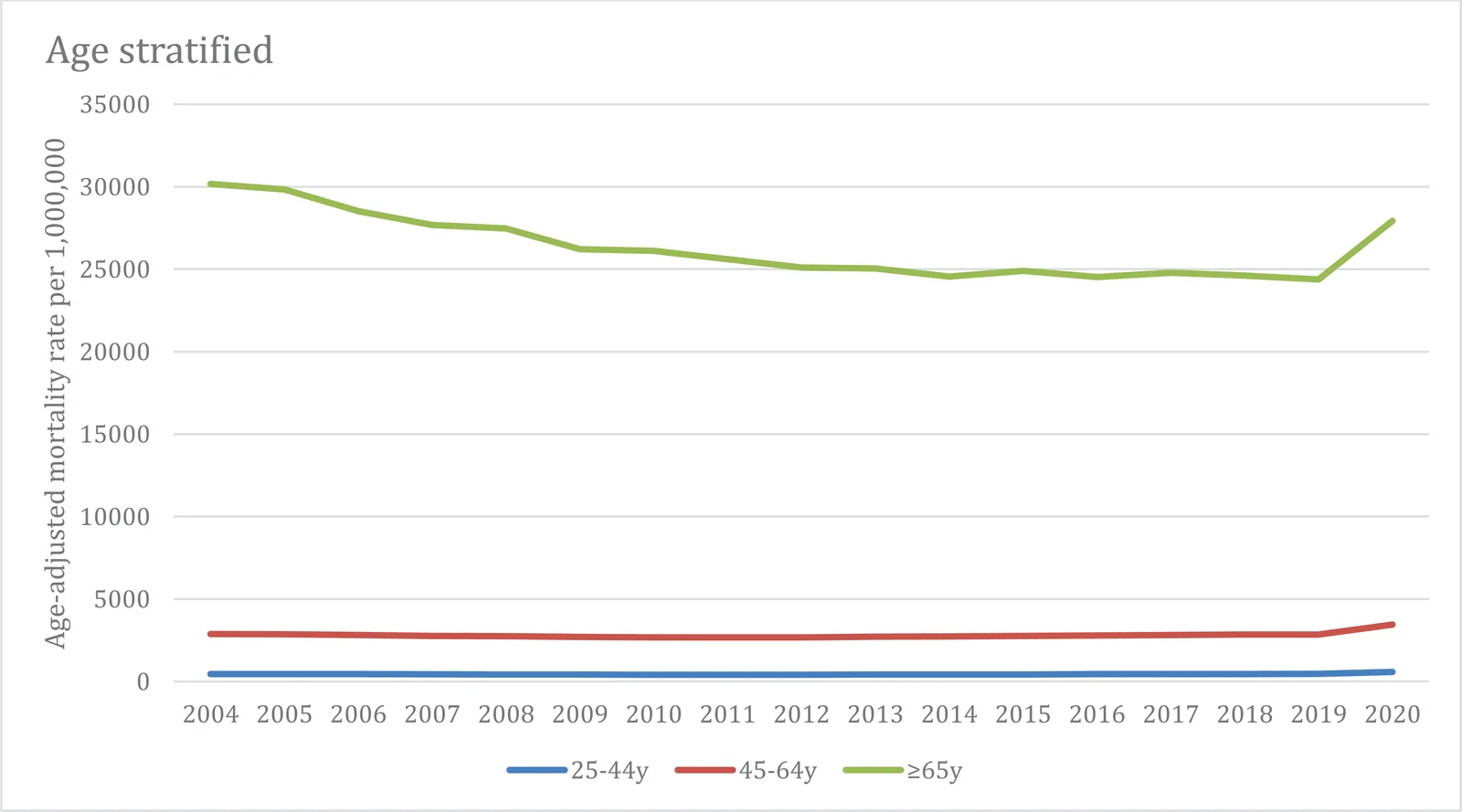
Cardiovascular disease-related AAMRs per 1,000,000 deaths stratified by age in the United States (2004 to 2020).

**Fig. (6a):**
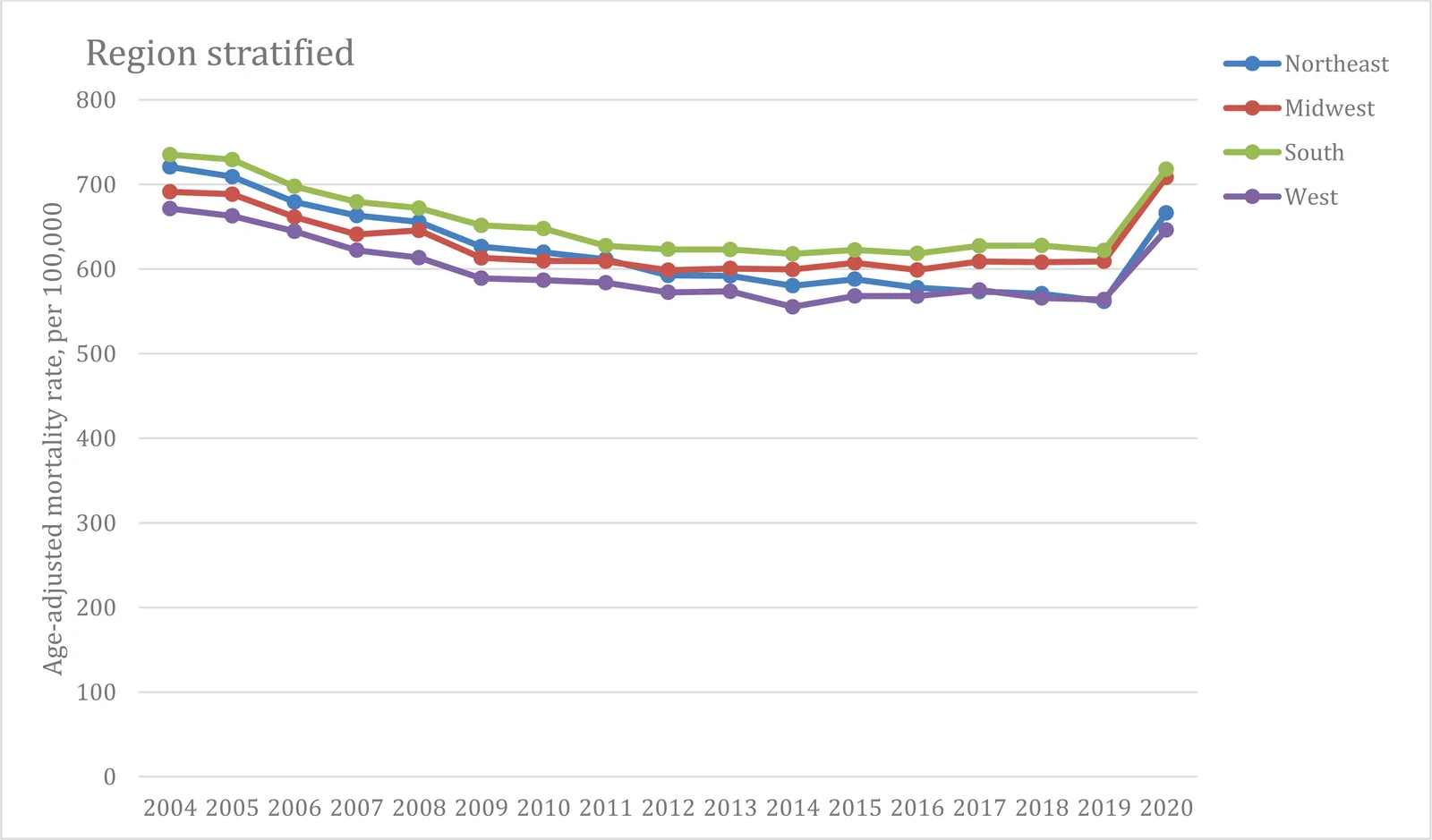
Cardiovascular disease-related AAMRs per 1,000,000, stratified by regions in the United States (2004 to 2020).

**Fig. (6b):**
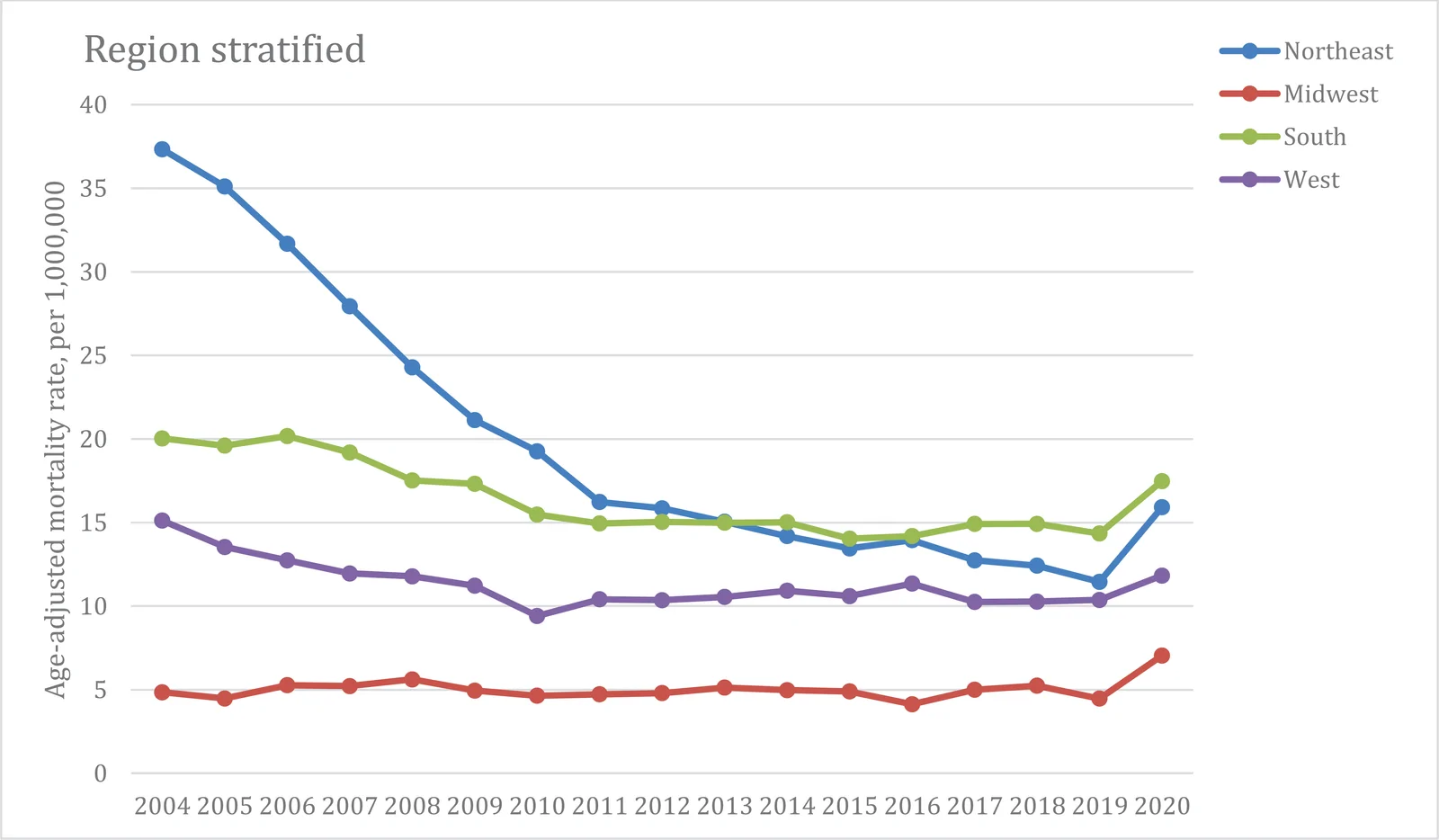
Cardiovascular disease with HIV-related AAMRs per 1,000,000, stratified by regions in the United States (2004 to 2020).

**Fig. (7a):**
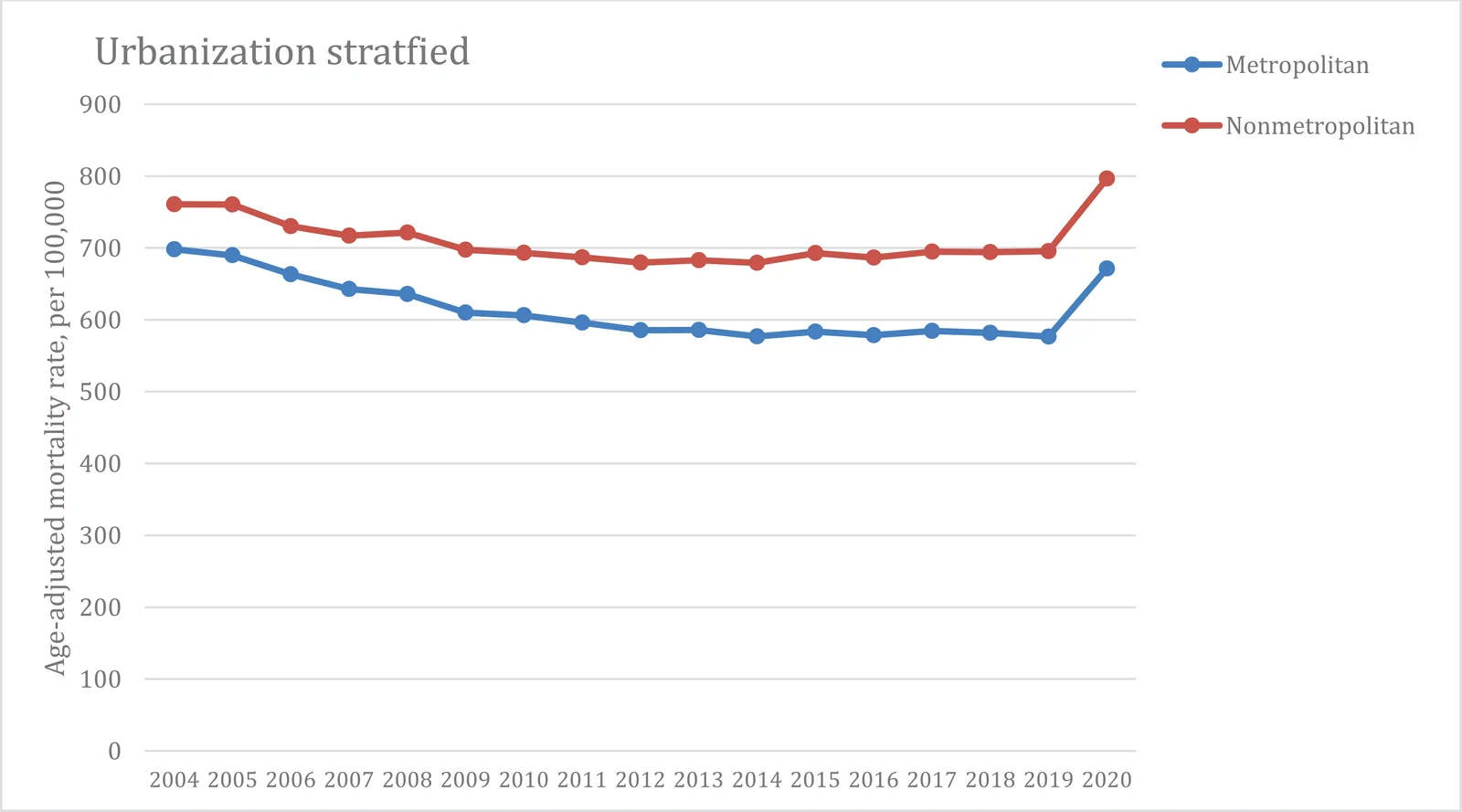
Cardiovascular disease-related AAMRs per 1,000,000, stratified by urbanization in the United States (2004 to 2020).

**Fig. (7b):**
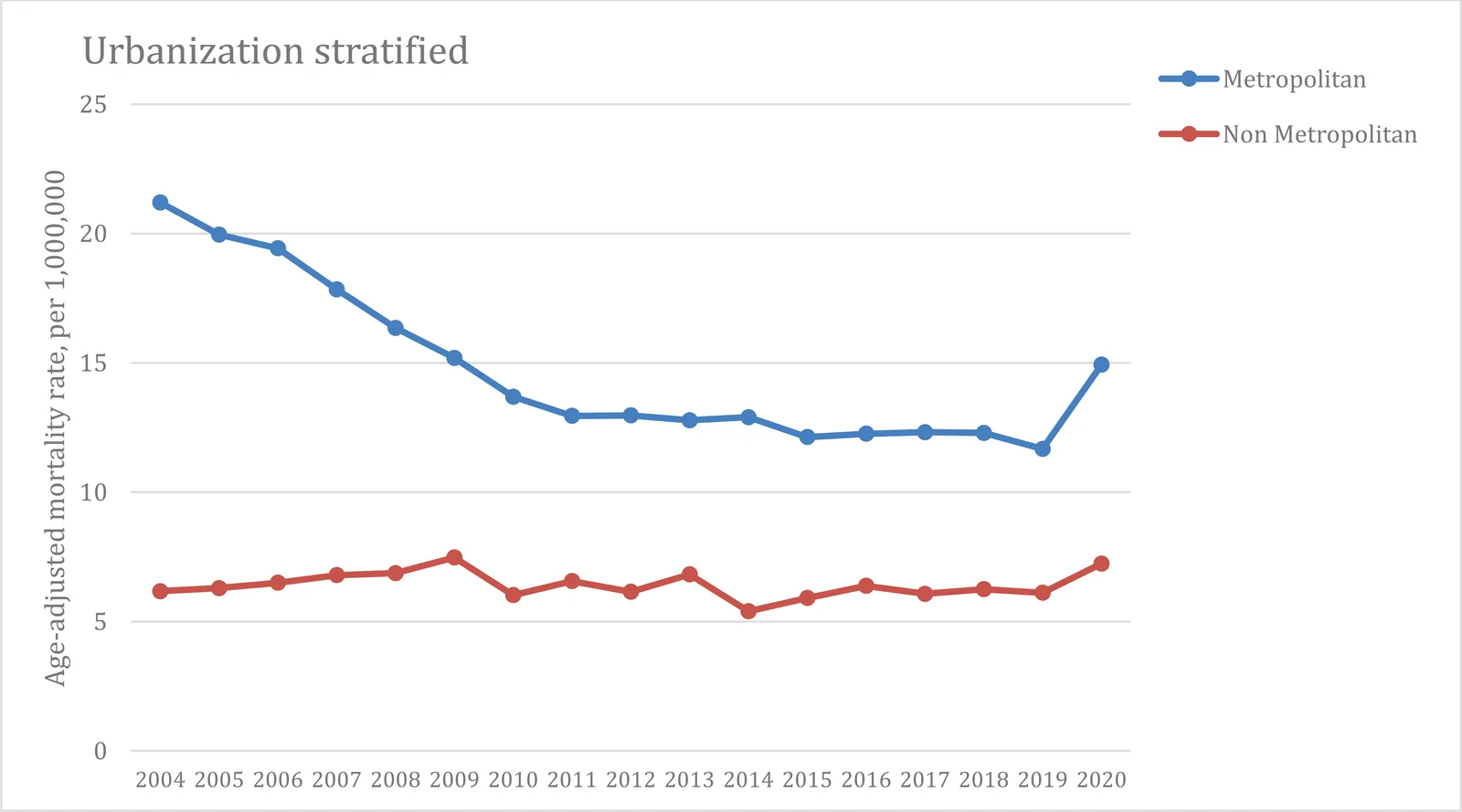
Cardiovascular disease with HIV-related AAMRs per 1,000,000, stratified by urbanization in the United States (2004 to 2020).

**Fig. (8a):**
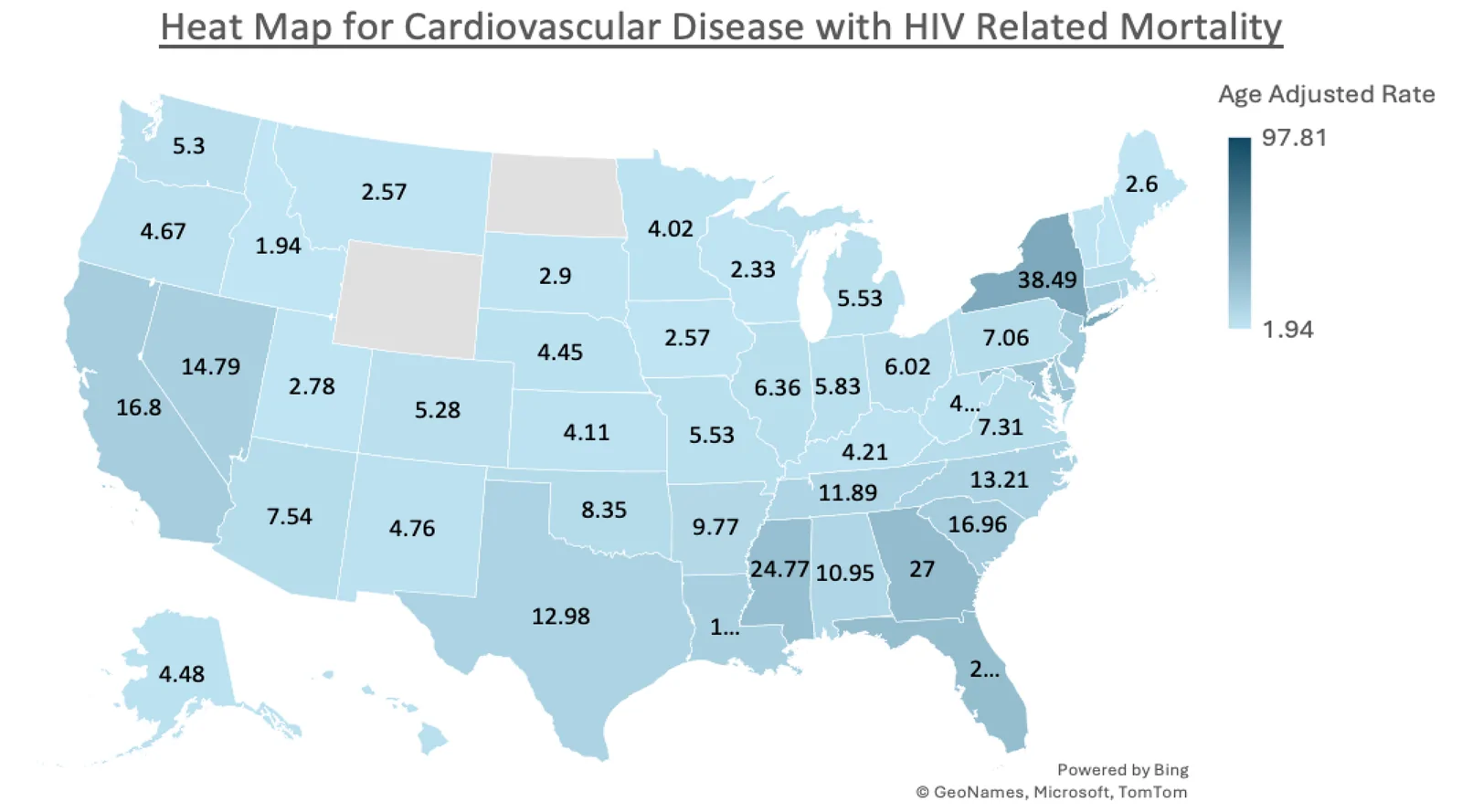
Cardiovascular disease with HIV-related AAMRs per 1,000,000, stratified by state (2004 to 2020 heatmap).

**Fig. (8b):**
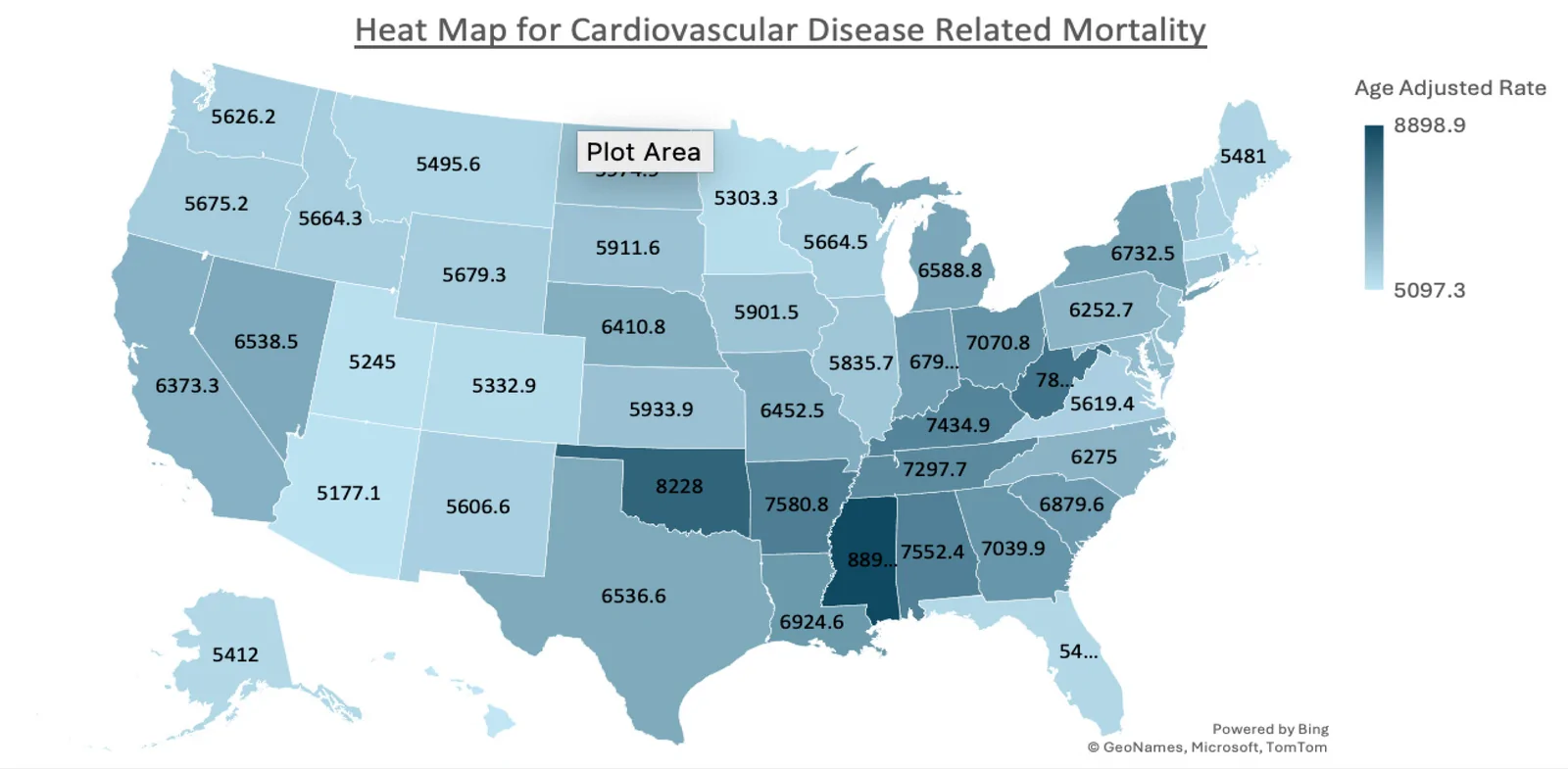
Cardiovascular disease-related AAMRs per 1,000,000, stratified by state (2004 to 2020 heatmap).

## Data Availability

The data supporting this study's findings are openly available in CDC WONDER at https://wonder.cdc.gov/, reference number N/A. All study data are publicly available at https://wonder.cdc.gov/.

## LIST OF ABBREVIATIONS

APC: Annual Percentage Change
AAPC: Average Annual Percentage Change
AAMR: Age-adjusted Mortality Rate
CD: Cluster of Differentiation
CI: Confidence Interval
COVID-19: Coronavirus Disease-2019
CVD: Cardiovascular Disease
ICD: International Classification of Diseases
HHD: Hypertensive Heart Disease
HIV: Human Immunodeficiency Virus
IRB: Institutional Review Board
NH: Non-Hispanic
PPV: Positive Predictive Value
US: United States

## STANDARDS OF REPORTING

STROBE guidelines were followed.
