# Supplementary material for: Trends and Disparities in Cardiovascular Mortality among HIV-Positive Adults in the United States (2004-2020): A CDC WONDER Database Analysis
Source: Curr HIV Res. 2026 Jan 9;24(2):110–25. doi: 10.2174/011570162X379326251026150021 (PMC13555775; doi:10.2174/011570162X379326251026150021)
Supplement: Supplementary file 1 [file CHIVR-24-2-110_SD1.pdf]

Supplementary Material

Trends and Disparities in Cardiovascular Mortality among HIV-Positive Adults in the United States (2004-2020): A CDC WONDER Database Analysis

Faizan Ahmed[1], Tehmasp Rehman Mirza[2], Sherif Eltawansy[3,*], Kainat Aman[4], Sonia Hurjkaliani[5], Adiesh Sood[6], Amnah Siddiqui[7], Yusra Junaid[5], Momina Siddiqui[8], Mushood Ahmed[8], Farman Ali[9] and Aman Ullah[10] and Nisar Asmi[11]

[1]Department of Internal Medicine, Duke University Hospital, Durham, NC, USA; [2]Department of Internal Medicine, Shalamar Medical and Dental College, Lahore, Pakistan; [3]Department of Internal Medicine, Jersey Shore University Medical Center, Neptune City, NJ, USA; [4]Department of Internal Medicine, Batterjee Medical College, Jeddah, Saudi Arabia; [5]Department of Internal Medicine, Dow University of Health Sciences, Karachi, Pakistan; [6]Department of Internal Medicine, Adesh Institute of Medical Sciences and Research (AIMSR), India; [7]Department of Internal Medicine, Karachi Medical and Dental College, Pakistan; [8]Department of Internal Medicine, Rawalpindi Medical University, Pakistan; [9]Department of Internal Medicine, Corewell Health William Beaumont University Hospital, Royal Oak, MI, USA; [10]Department of Medicine, Mississippi Academy of Sciences, Jackson, United States; [11]Department of Internal Medicine, University of North Carolina at Charlotte, NC, USA

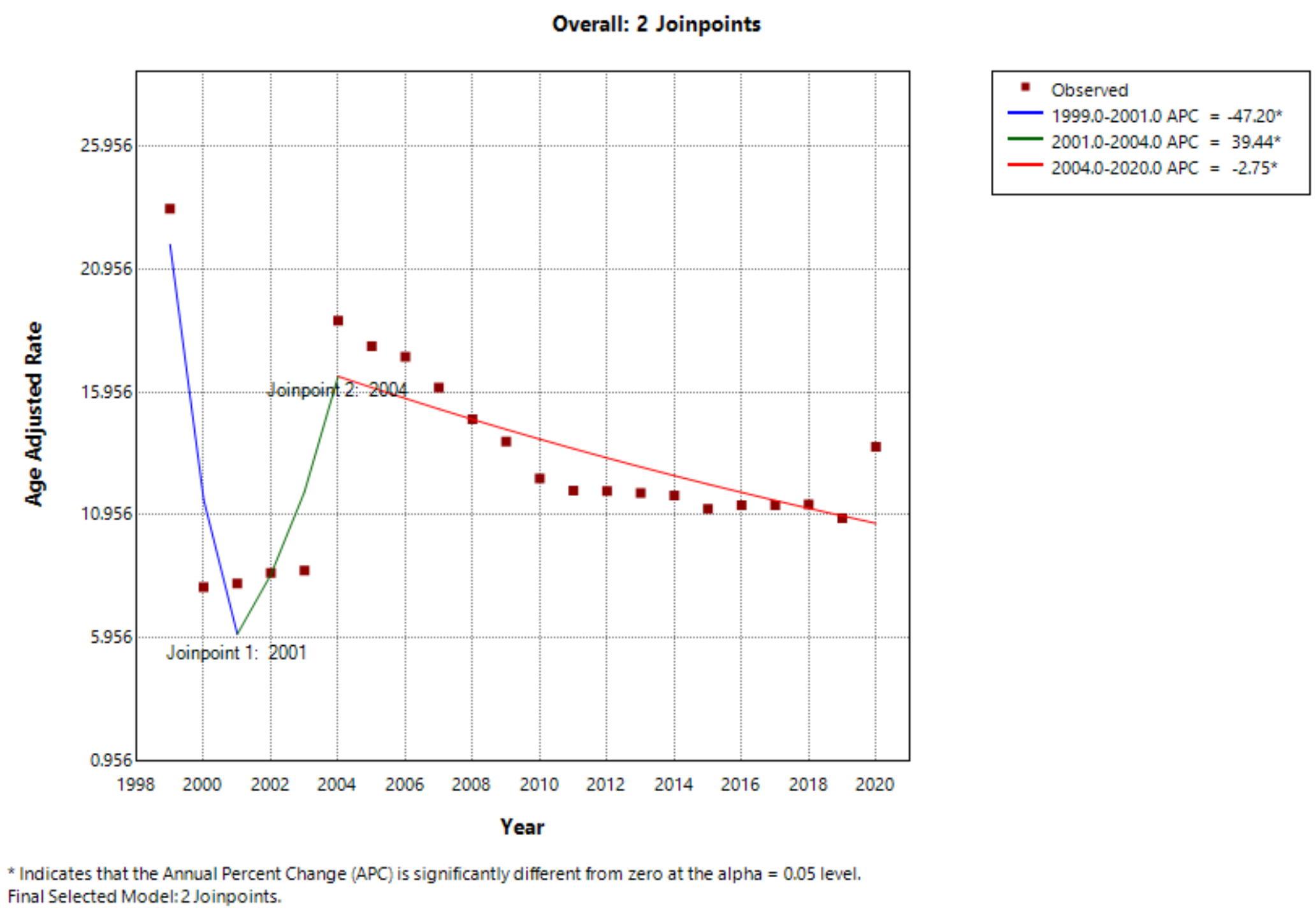

Supplementary Fig. (1a). Joinpoint analysis of CVD and HIV-related age-adjusted mortality rates from 1999-2020.

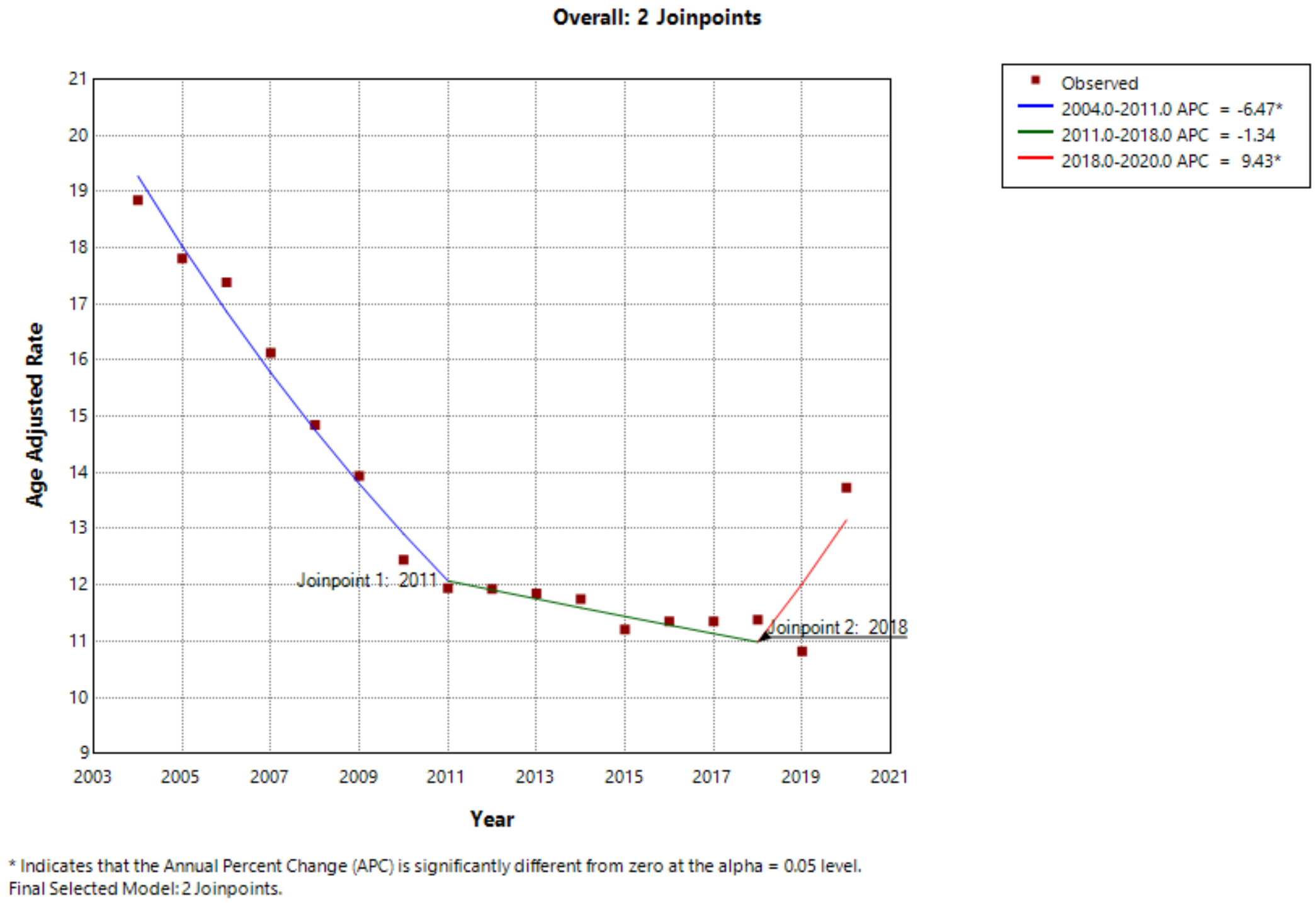

Supplementary Fig. (1b). Joinpoint analysis of CVD and HIV-related age-adjusted mortality rates from 2004-2020.

Figure. Trends in Cardiovascular Disease related and Cardiovascular Disease with HIV Patients Related Mortality Rates in the US, 2004 to 2020

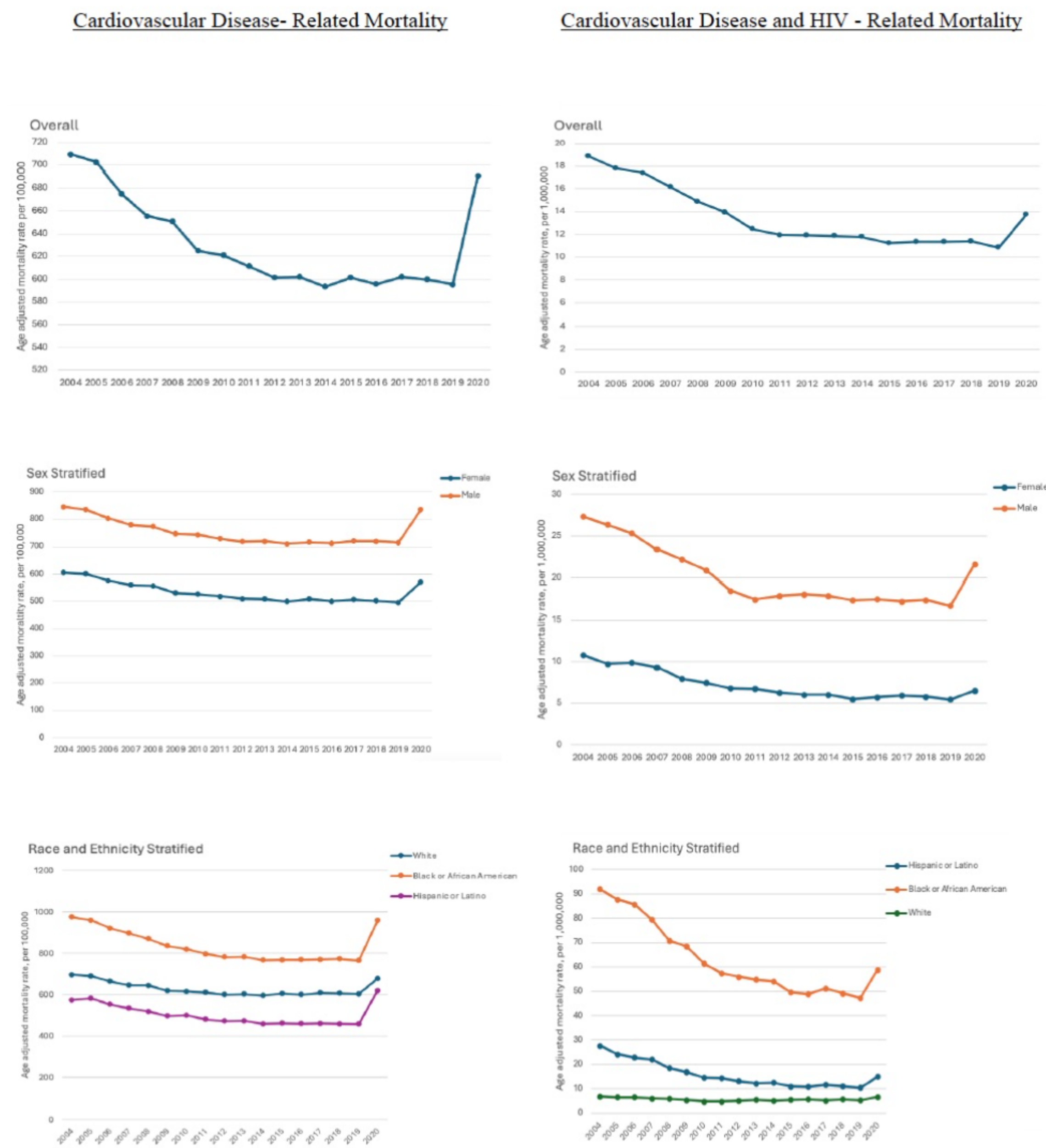

Supplementary Fig. (2). Trends in cardiovascular disease-related and cardiovascular disease with HIV-related mortality rates in the US (2004-2020) (overall and demographical linear trends and variations).

Figure. Trends in Cardiovascular Disease related and Cardiovascular Disease with HIV Related Mortality Rates in the US, 2004 to 2020

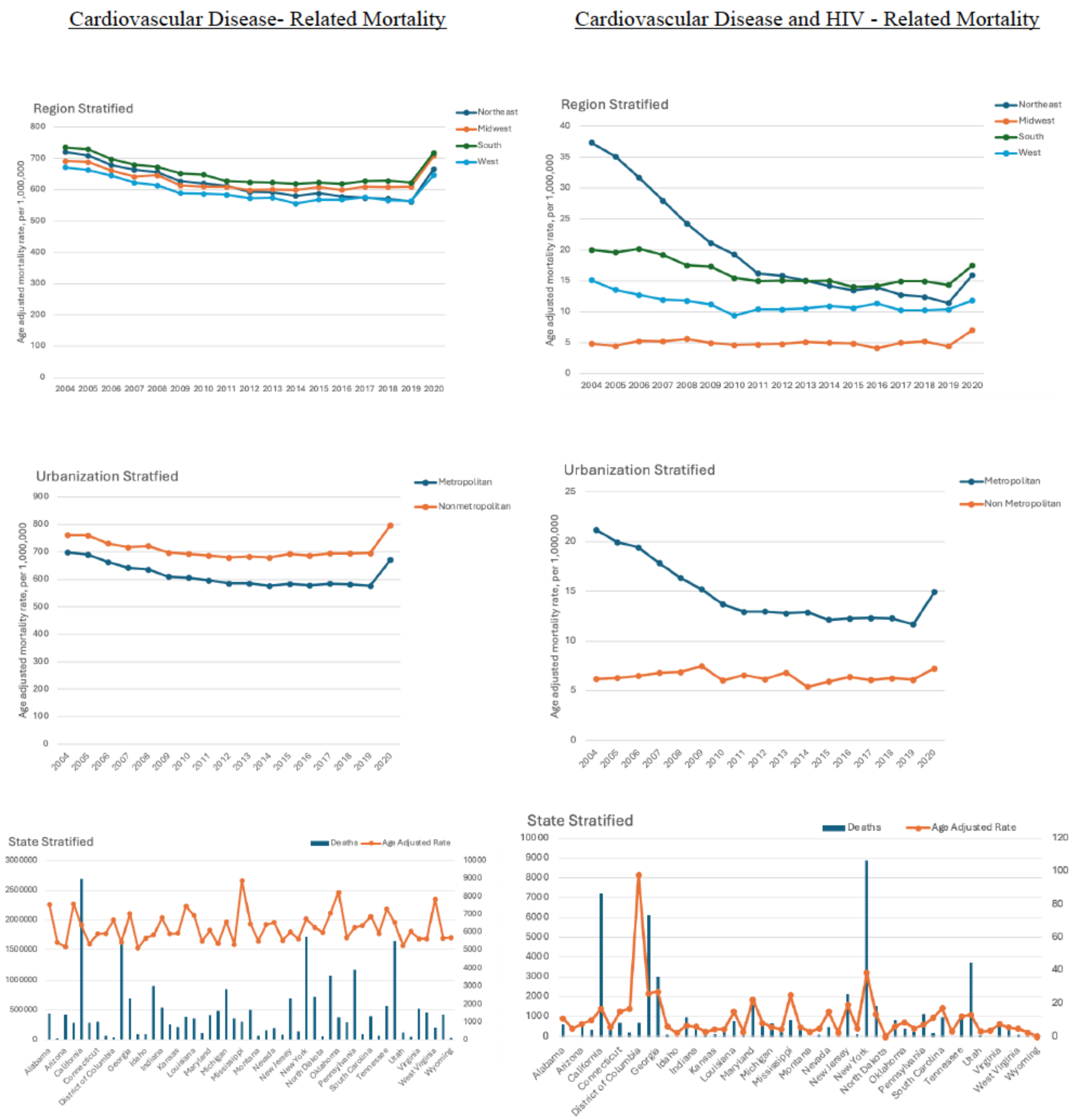

Supplementary Fig. (3). Trends in cardiovascular disease-related and cardiovascular disease with HIV-related mortality rates in the US (2004-2020) (geographical variations and trends).

Figure. Trends in Cardiovascular Disease and Cardiovascular Disease among HIV Patients Related Mortality Rates in the US, 2004 to 2020

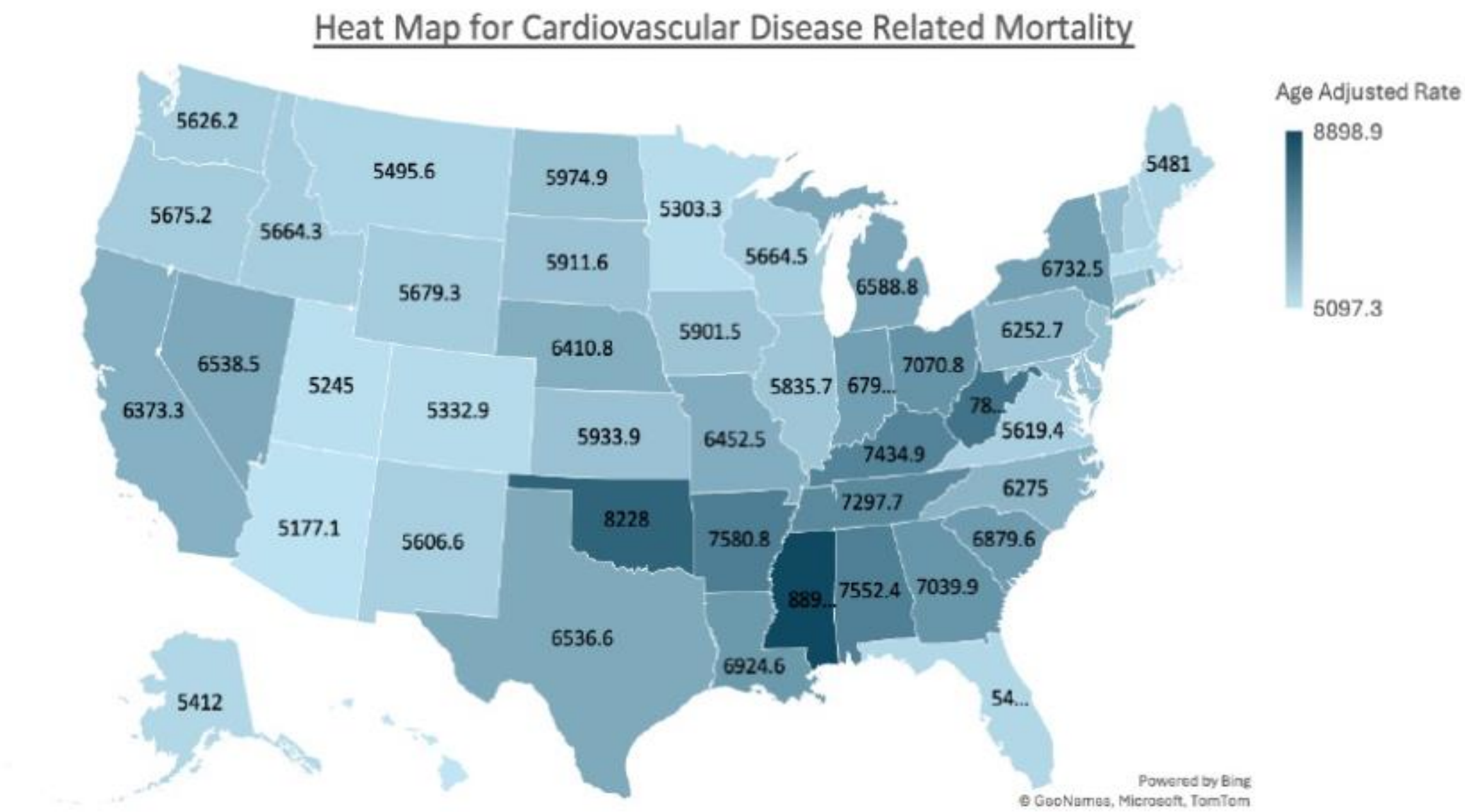

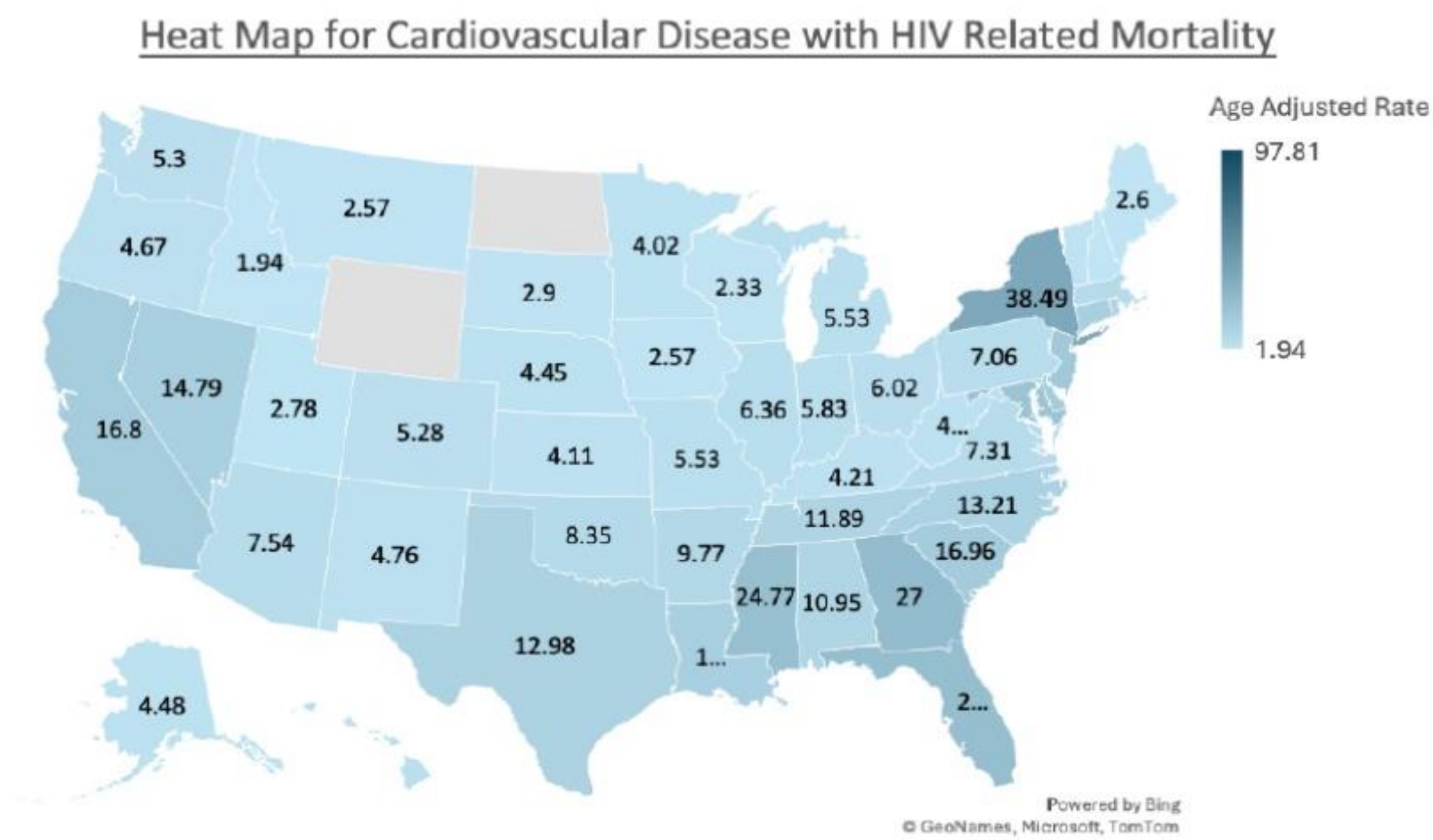

Supplementary Fig. (4). Trends in cardiovascular disease-related and cardiovascular disease with HIV-related mortality rates in the US (2004-2020) (state heatmaps).

Supplementary Table 1. Cardiovascular disease with HIV-related deaths, stratified by sex and race, in adults in the United States (2004 to 2020).

Deaths									
Year	Overall	Men	Women	White	Black or African American	Asian or Pacific Islander	American Indian or Alaska Native	Hispanic or Latino	Population
2004	3597	2560	1037	939	2023	17	22	554	190205384
2005	3472	2521	951	911	1970	27	26	512	192551384
2006	3440	2456	984	923	1955	28	12	498	195019359
2007	3241	2304	937	855	1850	17	10	491	197403777
2008	3049	2233	816	858	1677	37	18	433	199795090
2009	2920	2140	780	814	1652	17	15	403	202107016
2010	2668	1938	730	730	1516	33	Suppressed	357	203891983
2011	2616	1881	735	750	1453	19	14	362	206592936
2012	2645	1945	700	785	1445	22	15	343	208826037
2013	2671	1978	693	859	1430	20	18	319	211085314
2014	2686	1997	689	825	1438	16	12	353	213809280
2015	2634	1985	649	907	1340	22	15	319	216553817
2016	2707	2022	685	935	1360	32	23	322	218641417
2017	2782	2057	725	888	1457	25	19	353	221447331
2018	2790	2084	706	960	1401	32	16	350	223311190
2019	2703	2030	673	919	1379	25	18	340	224981167
2020	3511	2664	847	1175	1752	31	18	487	226635013
Total	50132	36795	13337	15033	27098	420	271	6796	3552857495

Supplementary Table 2. Average annual percent change (AAPC) of cardiovascular disease-related and cardiovascular disease with HIV-related age-adjusted mortality rates per 1,000,000 in older adults in the United States (2004 to 2020).

Cardiovascular disease		Cardiovascular disease with HIV	
Year interval	AAPC (95% CI)	Year interval	AAPC (95% CI)
Overall			
2004 to 2020	-0.3 (-0.74 to 0)	2004 to 2020	-2.36* (-3.13 to -1.91)
Men			
2004 to 2020	-0.23 (-0.72 to 0.14)	2004 to 2020	-1.89* (-2.7 to -1.38)
Women			
2004 to 2020	-0.54* (-0.96 to -0.26)	2004 to 2020	-3.64* (-4.52 to -2.87)

**25-44 years**			
2004 to 2020	1.34* (0.69 to 1.81)	2004 to 2020	-7.60* (-8.61 to -6.99)
**45-64 years**			
2004 to 2020	0.91* (0.34 to 1.37)	2004 to 2020	-1.93* (-2.61 to -1.41)
**≥65 years**			
2004 to 2020	-0.62* (-1.04 to -0.33)	2004 to 2020	6.63* (5.16 to 7.78)
**White**			
2004 to 2020	-0.29* (-0.65 to -0.05)	2004 to 2020	-0.90* (-1.57 to -0.19)
**Black or African American**			
2004 to 2020	-0.36 (-1.03 to 0.18)	2004 to 2020	-3.17* (-4.17 to -2.54)
**Hispanic or Latino**			
2004 to 2020	0.35 (-0.4 to 0.9)	2004 to 2020	-4.07* (-5.35 to -3.23)
**Non-metropolitan areas**			
2004 to 2020	0.15 (-0.25 to 0.42)	2004 to 2020	-0.21 (-1.13 to 0.73)
**Metropolitan area**			
2004 to 2020	-0.43* (-0.93 to -0.08)	2004 to 2020	-2.57* (-3.37 to -2.09)
**Northeast region**			
2004 to 2020	-0.50* (-1.2 to -0.13)	2004 to 2020	-5.78* (-6.77 to -5.26)
**Midwest region**			
2004 to 2020	-0.01 (-0.43 to 0.28)	2004 to 2020	1.66 (-0.02 to 2.64)
**South region**			
2004 to 2020	-0.32 (-0.78 to 0.01)	2004 to 2020	-1.42* (-2.08 to -0.84)
**West region**			
2004 to 2020	-0.40* (-0.78 to -0.09)	2004 to 2020	-1.76* (-2.37 to -1.1)

Abbreviations: AAPC = average annual percent change; N.H. = non-Hispanic; * indicates that the average annual percentage change (AAPC) is significantly different from zero at α = 0.05. AAMR = age-adjusted mortality rate.

Supplementary Table 3. Annual percent change (APC) of cardiovascular disease-related and cardiovascular disease with HIV-related age-adjusted mortality rates per 1,000,000 in older adults in the United States (2004 to 2020).

Cardiovascular disease		Cardiovascular disease with HIV	
**Year interval**	**APC (95% CI)**	**Year interval**	**APC (95% CI)**
**Overall**			
2004 to 2011	-2.24* (-5.17 to -1.31)	2004 to 2011	-6.47* (-10.20 to -4.97)
2011 to 2018	-0.35 (-1.65 to 1.36)	2011 to 2018	-1.34 (-5.00 to 1.09)
2018 to 2020	6.95* (2.57 to 9.45)	2018 to 2020	9.43* (1.13 to 14.37)
**Men**			
2004 to 2011	-2.19 (-5.32 to 0.27)	2004 to 2011	-6.08* (-10.46 to -4.41)
2011 to 2018	-0.30 (-1.93 to 1.51)	2011 to 2018	-0.92 (-4.80 to 1.67)
2018 to 2020	7.19* (2.29 to 9.85)	2018 to 2020	10.45* (1.65 to 15.69)
**Women**			
2004 to 2010	-2.59* (-5.39 to -1.66)	2004 to 2013	-6.77* (-10.05 to -5.25)
2010 to 2018	-0.68 (-1.65 to 0.77)	2013 to 2020	-0.54 (-2.08 to 7.70)
2018 to 2020	6.44* (2.26 to 8.64)	-	-
**25-44 years**			
2004 to 2011	-1.3 (-5.35 to 1.9)	2004 to 2007	-9.43* (-12.8 to -3.92)
2011 to 2018	1.25 (-1.2 to 3.76)	2007 to 2011	-15.51* (-19.35 to -9.01)
2018 to 2020	11.50* (4.8 to 15.31)	2011 to 2018	-5.72* (-11.2 to -2.61)
-	-	2018 to 2020	6.18 (-3.99 to 13.83)
**45-64 years**			
2004 to 2010	-1.34 (-5.1 to 1.8)	2004 to 2015	-3.26* (-5.51 to -2.53)
2010 to 2018	0.6 (-1.72 to 2.54)	2015 to 2020	1.06 (-1.5 to 7.74)
2018 to 2020	9.32* (3.55 to 12.32)	-	-
**≥65 years**			
2004 to 2011	-2.47* (-5.14 to -1.55)	2004 to 2018	4.60* (1.02 to 6.04)
2011 to 2018	-0.63 (-1.92 to 0.97)	2018 to 2020	21.95* (8.02 to 30.28)
2018 to 2020	6.12* (1.95 to 8.53)	-	-

**White**			
2004 to 2010	-2.23* (-4.48 to -1.38)	2004 to 2011	-4.95* (-8.00 to -3.04)
2010 to 2018	-0.24 (-1.16 to 0.86)	2011 to 2020	2.36* (0.92 to 4.40)
2018 to 2020	5.53* (1.89 to 7.46)	-	-
**Black or African American**			
2004 to 2011	-3.01 (-7.32 to 0.87)	2004 to 2011	-6.79* (-12.11 to -1.13)
2011 to 2018	-0.74 (-3.76 to 1.76)	2011 to 2018	-2.88 (-8.22 to 0.59)
2018 to 2020	11.02* (4.03 to 14.89)	2018 to 2020	9.46 (-0.95 to 15.67)
**Hispanic or Latino**			
2004 to 2018	-1.84* (-2.68 to -1.17)	2004 to 2011	-9.14* (-15.51 to -2.21)
2018 to 2020	17.12* (8.29 to 21.94)	2011 to 2018	-3.16 (-10.64 to 1.90)
-	-	2018 to 2020	15.87* (1.22 to 25.81)
**Non-metropolitan areas**			
2004 to 2011	-1.56* (-4.07 to -0.67)	2004 to 2020	-0.21 (-1.13 to 0.73)
2011 to 2018	0.11 (-1.11 to 1.59)	-	-
2018 to 2020	6.49* (2.48 to 8.70)	-	-
**Metropolitan area**			
2004 to 2011	-2.38 (-5.34 to 0.13)	2004 to 2011	-6.92* (-10.84 to -5.36)
2011 to 2018	-0.40 (-2.07 to 1.29)	2011 to 2018	-1.42 (-5.58 to 1.09)
2018 to 2020	6.58* (1.56 to 9.20)	2018 to 2020	9.72* (0.97 to 14.87)
**Northeast region**			
2004 to 2018	-1.76* (-2.47 to -1.31)	2004 to 2011	-11.35* (-14.91 to -9.86)
2018 to 2020	8.75* (1.26 to 12.18)	2011 to 2018	-4.80* (-8.84 to -1.63)
-	-	2018 to 2020	12.45* (0.42 to 19.79)
**Midwest region**			
2004 to 2010	-2.26* (-5.29 to -1.28)	2004 to 2018	-0.42 (-4.04 to 0.83)
2010 to 2018	-0.16 (-1.19 to 1.40)	2018 to 2020	17.48* (1.73 to 27.01)
2018 to 2020	7.67* (3.36 to 10.04)	-	-

**South region**			
2004 to 2011	-2.29* (-5.27 to -1.19)	2004 to 2015	-3.60* (-4.94 to -2.71)
2011 to 2018	-0.21 (-1.72 to 1.49)	2015 to 2020	3.53* (0.77 to 9.44)
2018 to 2020	6.46* (1.81 to 8.95)	-	-
**West region**			
2004 to 2010	-2.45* (-5.33 to -1.48)	2004 to 2010	-6.02* (-10.59 to -3.76)
2010 to 2018	-0.48 (-1.40 to 0.85)	2010 to 2020	0.88 (-0.24 to 2.59)
2018 to 2020	6.41* (2.53 to 8.63)	-	-

Abbreviations: APC = annual percent change; N.H. = non-Hispanic; * indicates that the annual percentage change (APC) is significantly different from zero at α = 0.05. AAMR = age-adjusted mortality rate.

Supplementary Table 4. Overall and sex-stratified cardiovascular disease-related and cardiovascular disease with HIV-related age-adjusted mortality rates per 1,000,000 in adults in the United States (2004-2020).

Age-adjusted rate (95% CI)						
	**Cardiovascular Disease-related**			**Cardiovascular Disease with HIV-related**		
**Year**	**Men**	**Women**	**Overall**	**Men**	**Women**	**Overall**
2004	8464 (8443-8485)	6063 (6049-6077)	7092 (7080-7104)	27.3 (26.3-28.4)	10.8 (10.1-11.4)	18.9 (18.2-19.5)
2005	8358 (8338-8379)	6011 (5997-6025)	7024 (7012-7035)	26.4 (25.3-27.4)	9.7 (9.1-10.4)	17.8 (17.2-18.4)
2006	8042 (8022-8062)	5762 (5749-5776)	6750 (6739-6762)	25.3 (24.3-26.4)	9.9 (9.3-10.5)	17.4 (16.8-18)
2007	7812 (7792-7831)	5592 (5579-5606)	6558 (6547-6569)	23.4 (22.5-24.4)	9.3 (8.7-9.9)	16.1 (15.6-16.7)
2008	7736 (7717-7755)	5558 (5545-5572)	6509 (6498-6520)	22.2 (21.3-23.1)	8.0 (7.4-8.5)	14.9 (14.3-15.4)
2009	7478 (7459-7496)	5303 (5290-5315)	6252 (6241-6263)	20.9 (20.0-21.8)	7.5 (6.9-8)	13.9 (13.4-14.5)
2010	7438 (7420-7456)	5261 (5248-5273)	6212 (6201-6222)	18.5 (17.7-19.4)	6.8 (6.3-7.3)	12.5 (12-12.9)
2011	7293 (7275-7310)	5188 (5175-5200)	6116 (6106-6127)	17.5 (16.7-18.3)	6.7 (6.2-7.2)	11.9 (11.5-12.4)
2012	7183 (7165-7200)	5090 (5078-5103)	6014 (6004-6025)	17.9 (17.1-18.7)	6.3 (5.6-6.7)	11.9 (11.5-12.4)
2013	7194 (7177-7211)	5084 (5072-5096)	6021 (6011-6031)	18.1 (17.3-18.8)	6.1 (5.6-6.5)	11.9 (11.4-12.3)

2014	7105 (7088-7122)	4998 (4986-5010)	5936 (5926-5946)	17.9 (17.1-18.7)	6.1 (5.6-6.5)	11.8 (11.3-12.2)
2015	7168 (7152-7185)	5079 (5067-5091)	6014 (6004-6024)	17.4 (16.6-18.2)	5.5 (5.1-5.9)	11.2 10.8-11.7)
2016	7136 (7119-7152)	5005 (4993-5017)	5960 (5950-5970)	17.5 (16.7-18.3)	5.7 (5.3-6.3)	11.4 (10.9-11.8)
2017	7208 (7191-7224)	5057 (5045-5068)	6022 (6013-6032)	17.3 (16.5-18.0)	6.0 (5.5-6.4)	11.4 (10.9-11.8)
2018	7195 (7179-7212)	5013 (5002-5025)	5997 (5988-6007)	17.4 (16.7-18.2)	5.8 (5.4-6.2)	11.4 (10.9-11.8)
2019	7156 (7140-7172)	4966 (4955-4977)	5956 (5946-5965)	16.7 (16-17.5)	5.5 (5.0-5.9)	10.8 (10.4-11.2)
2020	8358 (8341-8375)	5708 (5695-5720)	6910 (6900-6920)	21.6 (20.8-22.5)	6.5 (6.1-7)	13.7 (13.3-14.2)

Supplementary Table 5. Cardiovascular disease-related deaths, stratified by sex and race, in adults in the United States (2004 to 2020).

Deaths									
**Year**	**Overall**	**Men**	**Women**	**White**	**Black or African American**	**Asian or Pacific Islander**	**American Indian or Alaska Native**	**Hispanic or Latino**	**Population**
2004	1349244	642376	706868	1095965	158859	23373	5652	62458	190205384
2005	1363870	650555	713315	1103043	160738	24819	6038	66799	192551384
2006	1338927	643218	695709	1079661	158230	25611	6009	66932	195019359
2007	1328579	641018	687561	1068392	158086	25893	6140	68186	197403777
2008	1346713	650975	695738	1083131	157618	27231	6243	69847	199795090
2009	1320188	646024	674164	1055943	156103	27949	6433	71018	202107016
2010	1334582	654730	679852	1065304	156534	28881	6758	73989	203891983
2011	1351755	664672	687083	1078190	157442	29642	7094	76648	206592936
2012	1361812	673910	687902	1079574	160120	31004	7223	79786	208826037
2013	1395118	695363	699755	1100306	165668	33198	7627	84565	211085314
2014	1406790	706844	699946	1104399	168124	33827	8109	87712	213809280
2015	1457516	732204	725312	1139278	173743	36606	8590	93299	216553817
2016	1473345	747294	726051	1144533	179540	38130	8852	97437	218641417
2017	1522191	774795	747396	1177561	185722	41313	9336	103301	221447331
2018	1548931	794240	754691	1192550	191721	42994	9652	107469	223311190
2019	1567590	808729	758861	1202814	195288	44376	9627	111585	224981167

2020	1847526	965325	882201	1361526	251417	57555	12824	159314	226635013
Total	24314677	12092272	12222405	19132170	2934953	572402	132207	1480345	3552857495

Supplementary Table 6.. Race-stratified cardiovascular disease with HIV-related age-adjusted mortality rates per 1,000,000 in adults in the United States (2004 to 2020).

Age-adjusted Rate (95% CI)					
**Year**	**White**	**Black or African American**	**Asian or Pacific Islander**	**American Indian or Alaska Native**	**Hispanic or Latino**
2004	6.8 (6.4-7.2)	92.0 (88.0-96.0)	Unreliable	15.2 (9.5-23.0)	27.7 (25.4-30.0)
2005	6.5 (6.0-6.9)	87.8 (83.9-91.7)	3.1 (2.0-4.5)	18.2 (11.9-26.7)	24.2 (22.0-26.3)
2006	6.5 (6.1-6.9)	85.7 (81.9-89.5)	3.2 (2.1-4.7)	Unreliable	22.8 (20.8-24.9)
2007	6.0 (5.6-6.4)	79.4 (75.7-83.0)	Unreliable	Unreliable	21.9 (19.9-24.0)
2008	5.9 (5.5-6.3)	70.8 (67.4-74.3)	3.9 (2.7-5.4)	Unreliable	18.5 (16.7-20.3)
2009	5.4 (5.0-5.8)	68.4 (65.1-71.8)	Unreliable	Unreliable	16.8 (15.1-18.5)
2010	4.8 (4.4-5.1)	61.4 (58.3-64.6)	3.1 (2.1-4.4)	Suppressed	14.5 (13.0-16.1)
2011	4.7 (4.4-5.1)	57.4 (54.4-60.4)	Unreliable	Unreliable	14.4 (12.8-15.9)
2012	5.1 (4.7-5.5)	56.0 (53.0-58.9)	1.9 (1.2-2.9)	Unreliable	13.1 (11.7-14.5)
2013	5.5 (5.1-5.9)	54.8 (51.9-57.7)	1.7 (1.0-2.6)	Unreliable	12.2 (10.8-13.6)
2014	5.1 (4.7-5.5)	54.1 (51.4-57.0)	Unreliable	Unreliable	12.5 (11.2-13.9)
2015	5.4 (5.1-5.8)	49.6 (46.9-52.4)	1.7 (1.1-2.6)	Unreliable	11.0 (9.7-12.2)
2016	5.6 (5.3-6.0)	48.8 (46.2-51.5)	2.5 (1.7-3.5)	13.5 (8.4-20.4)	10.8 (9.6-12.0)
2017	5.2 (4.8-5.5)	51.2 (48.5-53.9)	1.8 (1.2-2.6)	Unreliable	11.7 (10.5-13.0)
2018	5.6 (5.3-6.0)	49.2 (46.5-51.8)	2.2 (1.5-3.1)	Unreliable	11.1 (9.9-12.3)
2019	5.3 (4.9-5.6)	47.3 (44.8-49.9)	1.7 (1.1-2.5)	Unreliable	10.5 (9.3-11.6)
2020	6.7 (6.3-7.1)	58.8 (56.0-61.7	2.2 (1.5-3.1)	Unreliable	14.9 (13.6-16.3)

Supplementary Table 7: Race-stratified cardiovascular disease-related age-adjusted mortality rates per 1,000,000 in adults in the United States (2004 to 2020).

Age-adjusted rate (95% CI)					
**Year**	**White**	**Black or African American**	**Asian or Pacific Islander**	**American Indian**	**Hispanic or Latino**
2004	6948 (6934-6961)	9740 (9691-9789)	4510 (4450-4570)	6551 (6368-6734)	5734 (5687-5782)
2005	6882 (6870-6895)	9588 (9540-9636)	4461 (4403-4518)	6689 (6508-6870)	5811 (5765-5857)
2006	6625 (6613-6638)	9188 (9141-9234)	4348 (4293-4403)	6464 (6289-6640)	5518 (5474-5562)
2007	6447 (6435-6460)	8953 (8907-8998)	4157 (4105-4210)	6419 (6246-6591)	5327 (5285-5369)
2008	6430 (6417-6442)	8691 (8646-8735)	4133 (4083-4184)	6218 (6053-6384)	5179 (5139-5220)
2009	6179 (6167-6191)	8342 (8299-8385)	4012 (3963-4060)	6165 (6003-6326)	4958 (4920-4996)
2010	6149 (6137-6161)	8187 (8145-8229)	3980 (3933-4027)	6352 (6190-6514)	4990 (4953-5028)
2011	6093 (6082-6105)	7952 (7911-7992)	3767 (3723-3811)	6273 (6118-6429)	4792 (4757-4827)
2012	5995 (5983-6006)	7804 (7765-7843)	3681 (3639-3722)	6067 (5919-6216)	4717 (4683-4751)
2013	6010 (5999-6022)	7816 (7777-7855)	3666 (3626-3707)	6087 (5943-6231)	4722 (4689-4755)
2014	5949 (5938-5961)	7659 (7622-7697)	3472 (3434-3509)	6158 (6017-6298)	4585 (4554-4616)
2015	6044 (6033-6056)	7667 (7630-7704)	3511 (3475-3548)	6236 (6098-6374)	4606 (4575-4637)
2016	5994 (5983-6005)	7683 (7647-7720)	3473 (3438-3509)	6147 (6014-6281)	4586 (4556-4616)
2017	6076 (6064-6087)	7695 (7659-7731)	3530 (3496-3565)	6264 (6132-6396)	4600 (4571-4629)
2018	6052 (6041-6063)	7719 (7684-7755)	3500 (3467-3534)	6129 (6002-6255)	4578 (4550-4607)
2019	6023 (6012-6034)	7642 (7607-7677)	3430 (3398-3462)	5918 (5796-6040)	4568 (4540-4596)
2020	6763 (6751-6775)	9576 (9538-9614)	4223 (4189-4258)	7541 (7406-7675)	6188 (6156-6219)

Supplementary Table 8. Age-stratified cardiovascular disease-related and cardiovascular disease with HIV-related age-adjusted mortality rates per 1,000,000 in adults in the United States (2004-2020).

Age-adjusted rate (95% CI)						
	Cardiovascular disease-related			Cardiovascular disease with HIV-related		
Year	Young (25-44 years)	Middle-aged (45-64 years)	Older (≥65 years)	Young (25-44 years)	Middle-aged (45-64 years)	Older (≥65 years)
2004	448 (444-453)	2882 (2870-2895)	30167 (30111-30223)	18 (17.1-19)	26.6 (25.4-27.8)	7.2 (6.3-8.1)
2005	447 (442-452)	2869 (2857-2881)	29842 (29786-29897)	15.4 (14.5-16.2)	26.8 (25.6-28)	7.7 (6.8-8.6)
2006	446 (441-450)	2823 (2811-2835)	28525 (28471-28578)	14.6 (13.7-15.4)	26.3 (25.2-27.5)	8.4 (7.4-9.3)
2007	432 (427-436)	2760 (2749-2772)	27687 (27635-27739)	13.2 (12.4-14)	25.2 (24.1-26.3)	7.2 (6.4-8.1)
2008	425 (421-430)	2747 (2736-2759)	27471 (27419-27523)	11.3 (10.6-12.1)	23 (21.9-24)	8.9 (8-9.9)
2009	428 (423-432)	2710 (2699-2722)	26216 (26166-26266)	9.3 (8.6-10)	22.7 (21.6-23.7)	9.5 (8.5-10.4)
2010	413 (408-417)	2674 (2663-2685)	26110 (26060-26160)	7.7 (7.1-8.3)	21.3 (20.3-22.3)	8.2 (7.3-9)
2011	415 (411-420)	2677 (2666-2688)	25610 (25562-25659)	6.4 (5.9-7)	21.1 (20.1-22.1)	8.9 (8-9.8)
2012	413 (408-417)	2671 (2660-2682)	25107 (25059-25154)	6.7 (6.1-7.3)	20.8 (19.8-21.8)	8.7 (7.8-9.6)
2013	420 (415-424)	2716 (2705-2727)	25042 (24995-25089)	5.8 (5.3-6.3)	20.7 (19.7-21.7)	10.6 (9.7-11.6)
2014	430 (425-435)	2735 (2724-2746)	24552 (24506-24598)	6 (5.5-6.5)	20.3 (19.3-21.3)	10.3 (9.4-11.3)
2015	431 (426-435)	2759 (2748-2770)	24907 (24861-24952)	5.1 (4.6-5.6)	18.8 (17.8-19.7)	12.3 (11.3-13.3)
2016	448 (443-452)	2797 (2786-2808)	24523 (24479-24568)	5.1 (4.6-5.6)	19.4 (18.4-20.3)	12.1 (11.1-13.1)
2017	453 (448-457)	2819 (2808-2830)	24792 (24748-24836)	4.6 (4.1-5.1)	19.4 (18.5-20.3)	13.2 (12.2-14.2)
2018	456 (452-461)	2844 (2833-2855)	24611 (24567-24654)	4.6 (4.1-5.1)	19 (18.1-20)	13.9 (12.9-14.9)
2019	461 (457-466)	2847 (2836-2858)	24382 (24339-24424)	4.2 (3.7-4.6)	17.8 (16.9-18.7)	14.3 (13.3-15.3)
2020	577 (572-583)	3446 (3434-3459)	27936 (27891-27981)	5.2 (4.8-5.7)	21.4 (20.4-22.4)	20.3 (19.2-21.5)

Supplementary Table 9. Cardiovascular disease-related and cardiovascular disease with HIV-related age-adjusted mortality rates per 1,000,000, stratified by Census region, in adults in the United States (2004-2020).

Age-Adjusted Rate (95% CI)								
-	Cardiovascular Disease-Related				Cardiovascular Disease with HIV-related			
Year	Northeast	Midwest	South	West	Northeast	Midwest	South	West
2004	7207 (7181-7234)	6913 (6889-6938)	7351 (7330-7372)	6715 (6689-6741)	37.3 (35.4-39.3)	4.9 (4.2-5.5)	20 (19-21.1)	15.1 (14-16.3)
2005	7092 (7066-7118)	6884 (6860-6908)	7294 (7274-7315)	6628 (6602-6653)	35.1 (33.2-37)	4.5 (3.9-5.1)	19.6 (18.6-20.6)	13.5 (12.5-14.6)
2006	6793 (6768-6819)	6615 (6592-6639)	6977 (6958-6997)	6446 (6422-6471)	31.7 (29.9-33.5)	5.3 (4.6-6)	20.2 (19.1-21.2)	12.7 (11.7-13.8)
2007	6632 (6607-6657)	6410 (6387-6433)	6794 (6775-6813)	6222 (6198-6245)	28 (26.3-29.6)	5.2 (4.5-5.9)	19.2 (18.2-20.2)	12 (11-13)
2008	6556 (6531-6580)	6457 (6434-6480)	6720 (6701-6738)	6134 (6110-6157)	24.3 (22.7-25.9)	5.6 (4.9-6.3)	17.5 (16.6-18.5)	11.8 (10.8-12.8)
2009	6265 (6241-6289)	6131 (6109-6153)	6516 (6497-6534)	5891 (5868-5914)	21.1 (19.7-22.6)	5 (4.3-5.6)	17.3 (16.4-18.3)	11.2 (10.3-12.2)
2010	6198 (6175-6222)	6097 (6075-6119)	6478 (6459-6496)	5868 (5846-5891)	19.3 (17.9-20.6)	4.6 (4-5.3)	15.5 (14.6-16.4)	9.4 (8.5-10.3)
2011	6117 (6094-6140)	6091 (6069-6113)	6276 (6259-6294)	5839 (5817-5861)	16.2 (15-17.5)	4.7 (4.1-5.4)	15 (14.1-15.8)	10.4 (9.5-11.3)
2012	5925 (5902-5948)	5987 (5966-6008)	6233 (6215-6250)	5724 (5703-5746)	15.9 (14.6-17.1)	4.8 (4.2-5.4)	15 (14.2-15.9)	10.4 (9.5-11.3)
2013	5920 (5897-5942)	6007 (5986-6029)	6231 (6214-6248)	5737 (5716-5758)	15.1 (13.9-16.2)	5.1 (4.5-5.8)	15 (14.1-15.8)	10.6 (9.7-11.5)
2014	5802 (5780-5824)	5994 (5973-6015)	6180 (6163-6196)	5554 (5534-5575)	14.2 (13-15.3)	5 (4.3-5.6)	15 (14.2-15.9)	10.9 (10-11.8)
2015	5880 (5857-5902)	6072 (6051-6094)	6226 (6210-6243)	5681 (5661-5702)	13.5 (12.4-14.6)	4.9 (4.3-5.5)	14 (13.2-14.9)	10.6 (9.7-11.5)
2016	5780 (5758-5802)	5989 (5968-6010)	6184 (6168-6201)	5680 (5660-5700)	14 (12.8-15.1)	4.1 (3.6-4.7)	14.2 (13.4-15)	11.4 (10.4-12.3)
2017	5733 (5711-5755)	6088 (6067-6109)	6274 (6258-6290)	5753 (5733-5773)	12.7 (11.7-13.8)	5 (4.4-5.6)	14.9 (14.1-15.7)	10.3 (9.4-11.1)
2018	5708 (5686-5729)	6080 (6059-6101)	6278 (6262-6294)	5657 (5637-5676)	12.4 (11.4-13.5)	5.2 (4.6-5.9)	14.9 (14.1-15.8)	10.3 (9.4-11.1)
2019	5618 (5596-5639)	6090 (6070-6111)	6219 (6203-6234)	5639 (5619-5658)	11.5 (10.4-12.5)	4.5 (3.9-5.1)	14.4 (13.6-15.1)	10.4 (9.5-11.2)

2020	6663 (6640-6686)	7083 (7061-7105)	7179 (7162-7196)	6461 (6441-6482)	50.9 (48.5-53.2)	7 (6.3-7.8)	17.5 (16.6-18.4)	11.8 (10.9-12.7)

Supplementary Table 10. Cardiovascular disease-related and cardiovascular disease with HIV-related age-adjusted mortality rates per 1,000,000, stratified by urban-rural classification, in adults in the United States (2004-2020).

Age-adjusted Rate (95% CI)				
	Cardiovascular Disease-related		Cardiovascular Disease with HIV-related	
Year	Metropolitan	Non-metropolitan	Metropolitan	Non-metropolitan
2004	6980 (6967-6993)	7609 (7580-7638)	21.2 (20.5-21.9)	6.2 (5.3-7.1)
2005	6897 (6885-6910)	7606 (7577-7635)	20.0 (19.3-20.7)	6.3 (5.4-7.2)
2006	6632 (6619-6644)	7303 (7275-7331)	19.4 (18.8-20.1)	6.5 (5.6-7.4)
2007	6427 (6415-6440)	7170 (7142-7198)	17.8 (17.2-18.5)	6.8 (5.9-7.7)
2008	6359 (6347-6371)	7213 (7185-7240)	16.4 (15.7-17.0)	6.9 (5.9-7.8)
2009	6100 (6088-6111)	6976 (6949-7003)	15.2 (14.6-15.8)	7.5 (6.5-8.5)
2010	6062 (6050-6073)	6931 (6904-6958)	13.7 (13.1-14.2)	6.0 (5.1-6.9)
2011	5962 (5951-5974)	6869 (6842-6895)	13.0 (12.4-13.5)	6.6 (5.6-7.5)
2012	5856 (5845-5867)	6796 (6770-6823)	13.0 (12.4-13.5)	6.2 (5.3-7.0)
2013	5859 (5848-5870)	6828 (6802-6854)	12.8 (12.3-13.3)	6.8 (5.9-7.8)
2014	5768 (5757-5778)	6791 (6765-6817)	12.9 (12.4-13.4)	5.4 (4.6-6.2)
2015	5835 (5824-5846)	6927 (6901-6953)	12.1 (11.6-12.6)	5.9 (5.1-6.8)
2016	5785 (5775-5796)	6867 (6841-6893)	12.3 (11.8-12.8)	6.4 (5.5-7.3)
2017	5845 (5835-5856)	6949 (6923-6975)	12.3 (11.8-12.8)	6.1 (5.2-6.9)
2018	5817 (5807-5828)	6943 (6918-6969)	12.3 (11.8-12.8)	6.3 (5.4-7.1)
2019	5767 (5756-5777)	6956 (6931-6982)	11.7 (11.2-12.1)	6.1 (5.3-7.0)
2020	6713 (6702-6724)	7967 (7940-7995)	14.9 (14.4-15.5)	7.2 (6.3-8.2)

Supplementary Table 11. Cardiovascular disease-related and cardiovascular disease with HIV-related deaths, stratified by place of death, in adults in the United States (2004-2020).

	Cardiovascular Disease-related	Cardiovascular Disease with HIV-related
Place of death	Deaths	
Medical facility - inpatient	8117976	27233
Medical facility - outpatient or ER	2102562	3822
Medical facility - dead on arrival	152929	261
Decedent's home	6473081	2228
Hospice facility	987087	5201
Nursing home/long-term care	5334015	1505
Other	1060328	415
Place of death unknown	86699	27233
Total	24314677	50132

Supplementary Table 12. Cardiovascular disease-related and cardiovascular disease with HIV-related age-adjusted mortality rates per 1,000,000 in adults in the United States (2004-2020).

Age-adjusted rate (95% CI)		
State	Cardiovascular Disease-related	Cardiovascular Disease with HIV-related
Alabama	7552.4 (7530.1-7574.8)	10.95 (10.06-11.85)
Alaska	5412 (5346.2-5477.8)	4.48 (3.05-6.36)
Arizona	5177.1 (5161.4-5192.7)	7.54 (6.9-8.17)
Arkansas	7580.8 (7552.7-7608.9)	9.77 (8.67-10.86)
California	6373.3 (6365.6-6380.9)	16.8 (16.41-17.19)
Colorado	5332.9 (5313.2-5352.6)	5.28 (4.7-5.87)
Connecticut	5890.4 (5869-5911.8)	15.03 (13.87-16.18)
Delaware	5920.7 (5876.8-5964.7)	16.75 (14.31-19.18)
District of Columbia	6675 (6612.8-6737.1)	97.81 (90.41-105.22)
Florida	5416.6 (5408.2-5424.9)	25.8 (25.13-26.46)
Georgia	7039.9 (7023.1-7056.7)	27 (26.03-27.97)
Hawaii	5097.3 (5065-5129.6)	5.69 (4.59-6.98)

Idaho	5664.3 (5629.6-5698.9)	1.94 (1.35-2.69)
Illinois	5835.7 (5823.6-5847.8)	6.36 (5.96-6.77)
Indiana	6790.4 (6772.2-6808.6)	5.83 (5.28-6.38)
Iowa	5901.5 (5878.6-5924.4)	2.57 (2.05-3.16)
Kansas	5933.9 (5908.8-5959)	4.11 (3.41-4.81)
Kentucky	7434.9 (7411.5-7458.3)	4.21 (3.64-4.78)
Louisiana	6924.6 (6901.8-6947.3)	14.94 (13.88-16.01)
Maine	5481 (5448.2-5513.9)	2.6 (1.89-3.5)
Maryland	6090.7 (6072.2-6109.3)	22.08 (20.98-23.18)
Massachusetts	5353 (5337.7-5368.2)	7.97 (7.35-8.59)
Michigan	6588.8 (6574.7-6602.9)	5.53 (5.1-5.96)
Minnesota	5303.3 (5285.9-5320.7)	4.02 (3.53-4.52)
Mississippi	8898.9 (8867.2-8930.5)	24.77 (23.03-26.5)
Missouri	6452.5 (6434.5-6470.4)	5.53 (4.97-6.09)
Montana	5495.6 (5456.1-5535.1)	2.57 (1.72-3.69)
Nebraska	6410.8 (6378.7-6442.9)	4.45 (3.57-5.48)
Nevada	6538.5 (6509-6568.1)	14.79 (13.43-16.14)
New Hampshire	5527.5 (5492.3-5562.6)	2.18 (1.52-3.03)
New Jersey	5991.3 (5977.1-6005.5)	19.08 (18.25-19.9)
New Mexico	5606.6 (5577-5636.2)	4.76 (3.88-5.64)
New York	6732.5 (6722.3-6742.6)	38.49 (37.68-39.3)
North Carolina	6275 (6260.4-6289.6)	13.21 (12.54-13.88)
North Dakota	5974.9 (5925.7-6024.1)	Unreliable (0.86-2.76)
Ohio	7070.8 (7057.4-7084.2)	6.02 (5.6-6.44)
Oklahoma	8228 (8201.7-8254.4)	8.35 (7.48-9.21)
Oregon	5675.2 (5654.5-5695.9)	4.67 (4.05-5.28)
Pennsylvania	6252.7 (6241.3-6264.1)	7.06 (6.63-7.48)
Rhode Island	6359.7 (6319.2-6400.2)	11.31 (9.49-13.13)
South Carolina	6879.6 (6858-6901.2)	16.96 (15.85-18.06)

South Dakota	5911.6 (5866.7-5956.5)	2.9 (1.88-4.28)
Tennessee	7297.7 (7278.6-7316.8)	11.89 (11.1-12.69)
Texas	6536.6 (6526.6-6546.7)	12.98 (12.56-13.4)
Utah	5245 (5215.8-5274.2)	2.78 (2.18-3.5)
Vermont	6034.3 (5982.1-6086.4)	3.27 (2.17-4.72)
Virginia	5619.4 (5604.1-5634.8)	7.31 (6.77-7.85)
Washington	5626.2 (5609.8-5642.7)	5.3 (4.8-5.8)
West Virginia	7840.7 (7806.4-7874.9)	4.53 (3.63-5.44)
Wisconsin	5664.5 (5647.5-5681.5)	2.33 (1.97-2.7)
Wyoming	5679.3 (5621.2-5737.4)	Unreliable (0.83-2.98)

Supplementary Table 13. Cardiovascular disease-related age-adjusted mortality rates per 1,000,000, stratified by states, in adults in the United States (2004-2020), ranked according to percentile.

State	Age-adjusted Rate (95% CI)	Percentile	Rank
Mississippi	8898.9	98	1
Oklahoma	8228	96	2
West Virginia	7840.7	94	3
Arkansas	7580.8	92	4
Alabama	7552.4	90	5
Kentucky	7434.9	88	6
Tennessee	7297.7	87	7
Ohio	7070.8	85	8
Georgia	7039.9	83	9
Louisiana	6924.6	81	10
South Carolina	6879.6	79	11
Indiana	6790.4	77	12
New York	6732.5	75	13
District of Columbia	6675	73	14
Michigan	6588.8	71	15

Nevada	6538.5	69	16
Texas	6536.6	65	17
Missouri	6452.5	65	18
Nebraska	6410.8	63	19
California	6373.3	62	20
Rhode Island	6359.7	60	21
North Carolina	6275	58	22
Pennsylvania	6252.7	56	23
Maryland	6090.7	54	24
Vermont	6034.3	52	25
New Jersey	5991.3	50	26
North Dakota	5974.9	48	27
Kansas	5933.9	46	28
Delaware	5920.7	44	29
South Dakota	5911.6	42	30
Iowa	5901.5	40	31
Connecticut	5890.4	38	32
Illinois	5835.7	37	33
Wyoming	5679.3	35	34
Oregon	5675.2	33	35
Wisconsin	5664.5	31	36
Idaho	5664.3	29	37
Washington	5626.2	27	38
Virginia	5619.4	25	39
New Mexico	5606.6	23	40
New Hampshire	5527.5	21	41
Montana	5495.6	19	42
Maine	5481	17	43
Florida	5416.6	15	44

Alaska	5412	13	45
Massachusetts	5353	12	46
Colorado	5332.9	10	47
Minnesota	5303.3	8	48
Utah	5245	6	49
Arizona	5177.1	4	50
Hawaii	5097.3	2	51

Supplementary Table 14. Cardiovascular disease with HIV-related age-adjusted mortality rates per 1,000,000, stratified by states, in adults in the United States (2004-2020), ranked according to percentile.

State	Age-adjusted Rate (95% CI)	Percentile	Rank
District of Columbia	97.81	98	1
New York	38.49	96	2
Georgia	27	94	3
Florida	25.8	92	4
Mississippi	24.77	90	5
Maryland	22.08	88	6
New Jersey	19.08	86	7
South Carolina	16.96	84	8
California	16.8	82	9
Delaware	16.75	80	10
Connecticut	15.03	78	11
Louisiana	14.94	76	12
Nevada	14.79	74	13
North Carolina	13.21	72	14
Texas	12.98	70	15
Tennessee	11.89	68	16
Rhode Island	11.31	66	17
Alabama	10.95	64	18

Arkansas	9.77	62	19
Oklahoma	8.35	60	20
Massachusetts	7.97	58	21
Arizona	7.54	56	22
Virginia	7.31	54	23
Pennsylvania	7.06	52	24
Illinois	6.36	50	25
Ohio	6.02	46	26
Indiana	5.83	46	27
Hawaii	5.69	44	28
Michigan	5.53	42	29
Missouri	5.53	40	30
Washington	5.3	38	31
Colorado	5.28	36	32
New Mexico	4.76	34	33
Oregon	4.67	30	34
West Virginia	4.53	30	35
Alaska	4.48	28	36
Nebraska	4.45	26	37
Kentucky	4.21	24	38
Kansas	4.11	22	39
Minnesota	4.02	20	40
Vermont	3.27	18	41
South Dakota	2.9	16	42
Utah	2.78	14	43
Maine	2.6	12	44
Iowa	2.57	10	45
Montana	2.57	8	46
Wisconsin	2.33	6	47

New Hampshire	2.18	4	48
Idaho	1.94	2	49
Wyoming	Unreliable	-	-
North Dakota	Unreliable	-	-

STROBE CHECKLIST (FILLED)

Study Title: Temporal and Demographic Patterns of Mortality from Co-Occurring Heart Failure and Chronic Lower Respiratory Disease in the United States, 1999–2024

Study Type: Population-based observational (ecological, time-trend) study using CDC WONDER data (1999–2024)

Authors: Muhammad Hammad Chola et al.

STROBE Item	Recommendation	Location in Manuscript
1	Indicate study design in title/abstract	Title and Abstract – ‘Population-based study’ and Methods section 2.1
2	Explain scientific background and rationale	Introduction – Paragraphs 1–3
3	State specific objectives	End of Introduction – last paragraph
4	Present key elements of study design early	Methods 2.1 Study Design
5	Describe setting, locations, and relevant dates	Methods 2.1 and 2.4 (1999–2024, United States, CDC WONDER data)
6	Eligibility criteria and selection of participants	Methods 2.2–2.3
7	Clearly define outcomes, exposures, predictors, confounders	Methods 2.4; ICD-10 codes I11.0, I13.0, I13.2, I50, J40–J47
8	Sources of data and details of methods of assessment	Methods 2.4 Data Extraction
9	Describe efforts to address potential bias	Discussion – Limitations paragraph
10	Explain how study size was arrived at	Methods 2.5 Statistical Analysis (nationwide registry data, full population)
11	Explain handling of quantitative variables	Methods 2.5 Statistical Analysis
12	Describe all statistical methods including confounder control	Methods 2.5 Statistical Analysis (Joinpoint regression, AAMRs, APC, AAPC)
13	Report numbers at each study stage	Results 3.0 and Table 1
14	Give characteristics of study participants	Results 3.5, Table 1 (Demographics)
15	Report numbers of outcome events	Results 3.0 (1,334,449 deaths reported)
16	Give unadjusted and adjusted estimates with precision	Results 3.1–3.5 (AAMR with 95% CI)
17	Report other analyses (subgroups/interactions)	Results 3.2–3.5 (sex, race, region, urban-rural)
18	Summarise key results with reference to objectives	Discussion – Opening paragraphs
19	Discuss limitations and potential bias	Discussion – Limitations paragraph
20	Give cautious overall interpretation considering evidence	Discussion – Final paragraphs before Conclusion
21	Discuss generalisability (external validity)	Discussion – Geographic and demographic generalisation paragraph
22	Give source of funding and role of funders	End of Manuscript – Financial support: none
